# Comparative genomic analyses reveal the genetic basis of the yellow-seed trait in *Brassica napus*

**DOI:** 10.1038/s41467-023-40838-1

**Published:** 2023-08-25

**Authors:** Cunmin Qu, Meichen Zhu, Ran Hu, Yongchao Niu, Si Chen, Huiyan Zhao, Chengxiang Li, Zhen Wang, Nengwen Yin, Fujun Sun, Zhiyou Chen, Shulin Shen, Guoxia Shang, Yan Zhou, Xingying Yan, Lijuan Wei, Liezhao Liu, Bin Yi, Jinmin Lian, Jiang Li, Zhanglin Tang, Ying Liang, Xinfu Xu, Rui Wang, Jiaming Yin, Huafang Wan, Hai Du, Wei Qian, Yourong Chai, Qingyuan Zhou, Yajun He, Silin Zhong, Xiao Qiu, Hao Yu, Hon-Ming Lam, Kun Lu, Fuyou Fu, Jiana Li

**Affiliations:** 1 Engineering Research Center of South Upland Agriculture, Ministry of Education, College of Agronomy and Biotechnology, Southwest University, Chongqing, China; 2 Integrative Science Center of Germplasm Creation in Western China (CHONGQING) Science City and Southwest University, Chongqing, China; 3 Academy of Agricultural Sciences, Southwest University, Chongqing, China; 4 The State Key Laboratory of Agrobiotechnology and School of Life Sciences, The Chinese University of Hong Kong, Hong Kong SAR, China; 5 Department of Biological Sciences, Faculty of Science, National University of Singapore, Singapore, Singapore; 6 National Key Laboratory Breeding Base for Innovation and Utilization of Plateau Crop Germplasm, Academy of Agricultural and Forestry Sciences, Qinghai University, Xining, Qinghai China; 7 National Key Laboratory of Crop Genetic Improvement, National Center of Rapeseed Improvement in Wuhan, Huazhong Agricultural University, Wuhan, China; 8 Biozeron Shenzhen, Inc, Shenzhen, China; 9 Department of Food and Bioproduct Sciences, University of Saskatchewan, Saskatoon, Canada; 10 Temasek Life Sciences Laboratory, National University of Singapore, Singapore, Singapore; 11 Agriculture and Agri-Food Canada, Saskatoon Research Centre, 107 Science Place, Saskatoon, Canada

**Keywords:** Plant breeding, Plant molecular biology, Agricultural genetics, Comparative genomics

## Abstract

Yellow-seed trait is a desirable breeding characteristic of rapeseed (*Brassica napus*) that could greatly improve seed oil yield and quality. However, the underlying mechanisms controlling this phenotype in *B. napus* plants are difficult to discern because of their complexity. Here, we assemble high-quality genomes of yellow-seeded (GH06) and black-seeded (ZY821). Combining in-depth fine mapping of a quantitative trait locus (QTL) for seed color with other omics data reveal *BnA09MYB47a*, encoding an R2R3-MYB-type transcription factor, as the causal gene of a major QTL controlling the yellow-seed trait. Functional studies show that sequence variation of BnA09MYB47a underlies the functional divergence between the yellow- and black-seeded *B. napus*. The black-seed allele BnA09MYB47a^ZY821^, but not the yellow-seed allele BnA09MYB47a^GH06^, promotes flavonoid biosynthesis by directly activating the expression of *BnTT18*. Our discovery suggests a possible approach to breeding *B. napus* for improved commercial value and facilitates flavonoid biosynthesis studies in *Brassica* crops.

## Abstract (web summary)

Yellow-seed trait is preferred in rapeseed breeding as it can greatly improve seed oil yield and quality. Here, the authors assemble the genome of two rapeseed lines with yellow-seed and black-seed phenotypes, and clone an R2R3-MYB-type transcription factor encoding gene as a key regulator of seed color.

## Introduction

Rapeseed (*Brassica napus* L.; AACC, 2n = 38), a relatively recent allotetraploid (<7500 years ago), originated from natural hybridization between its diploid progenitors *B. rapa* (AA, 2n = 20) and *B. oleracea* (CC, 2n = 18)^1^. *B*. *napus* is an important oilseed crop that supplies vegetable oil for human consumption and biodiesel production, and is a source of protein-rich feed for livestock^2^. The yellow-seed trait is particularly desirable in *B*. *napus*, as it is associated with higher oil and protein yields and improved seed quality^3,4^. Furthermore, this trait is associated with more transparent oil, lower fiber content, desirable aroma, and higher nutrients content^5,6^ (Supplementary Fig. 1), and facilitates industrial processing, as yellow seeds lack the pigment deposits found in black/dark *B. napus* that interfere with processing.

Although *B*. *napus* is the most important *Brassica* oilseed species, natural rapeseed germplasms with the yellow-seed trait have not been identified. Yellow-seeded *B. napus* lines were created by interspecific hybridization with related species, for example, between *B*. *rapa* and *B*. *oleracea*, *Brassica juncea* and *Brassica carinata*, *B*. *napus*, and *Sinapis alba*, and *Brassica alboglabra* and *B*. *rapa*^7–10^. However, as the yellow-seed trait is a quantitative trait that is also affected by environmental factors, obtaining stable commercial *B*. *napus* varieties with yellow seeds is challenging. In the 1990s, we developed the yellow-seeded genetic resource of *B. oleracea* from *B*. *oleracea* var. *aceaphala* by mutation and subsequently bred commercial yellow-seeded *B. napus* varieties through interspecific hybridization between *B*. *rapa* and *B*. *juncea* (Supplementary Fig. 1). The widely cultivated yellow-seeded elite *B*. *napus* variety GH06 was derived from the interspecific hybridization between the *Brassica* species (A and C genomes)^3,11^, and selected as the breeding parent of Yu-yellow No. 1 (China National certified No. 2003024, Supplementary Fig. 1), a popular cultivar in the Yangtze River area and southwestern China with a cultivation area exceeding 10 million acres^11^. Recently, several more yellow-seeded varieties (e.g., Yu-yellow No. 2, Yu-yellow No. 4, and Yuyou28) and many more yellow-seeded germplasm resources have been developed (Supplementary Fig. 1), serving as the leading *B. napus* cultivated varieties in southwestern China (http://www.moa.gov.cn). Therefore, understanding the mechanism controlling the yellow-seed trait in GH06 is imperative for the oilseed industry, which can provide a theoretical basis for breeding and speed up the breeding process.

Given its important commercial value, exploring the mechanism of the yellow-seed trait has been a hotspot in the past three decades. However, the inheritance of the yellow-seed trait in *B. napus* is complex. The candidate genes controlling seed color in *B. napus* vary depending on the genetic background, and the trait usually requires three or four independent alleles^12–15^. The effects of quantitative trait loci (QTLs) controlling seed color in diverse populations have been analyzed using classical genetic tools^15–23^. A major QTL with strong effects on seed color has been widely identified on chromosome A09 in different studies^19,22–24^. Our previous results showed that the yellow-seed trait in GH06 is mainly controlled by a few dominant genes, which are subjected to epistatic and environmental effects^3,11^, and the candidate QTL region was also identified using two recombinant inbred line (RIL) populations derived from the same female parent, GH06^19^. However, the causal genes of the yellow-seed trait within these loci have not been identified or characterized to date.

In this study, we further characterize the major QTL on chromosome A09 (explaining >40% of the phenotypic variance) using a RIL population derived from a cross between the female parent GH06 (yellow-seeded) and the male parent ZY821 (black-seeded), both of which are elite *B*. *napus* varieties grown in China^25^ that are often used as control cultivars^26,27^. To dissect the genetic mechanism underlying the yellow-seed trait, we first generate high-quality reference genomes of GH06 and ZY821 using PacBio, high‐throughput chromosome conformation capture (Hi-C), and RNA sequencing (RNA-seq) data. Combining the de novo assembled genome sequences with QTL sequencing and map-based cloning reveal *BnA09MYB47a*, encoding an R2R3-MYB-type transcription factor, as the causal gene of a major QTL controlling the yellow-seed trait. We further demonstrate that BnA09MYB47a regulates flavonoid biosynthesis in *B*. *napus* seed coats. The results provide insights into the transcriptional regulation of flavonoid biosynthesis genes in polyploid crops and highlight the potential application of molecular mechanism studies on yellow seeds for breeding oil-rich varieties in *Brassica* crops.

## Results

### De novo assembly and annotation of the GH06 and ZY821 genomes

**Fig. 1:**
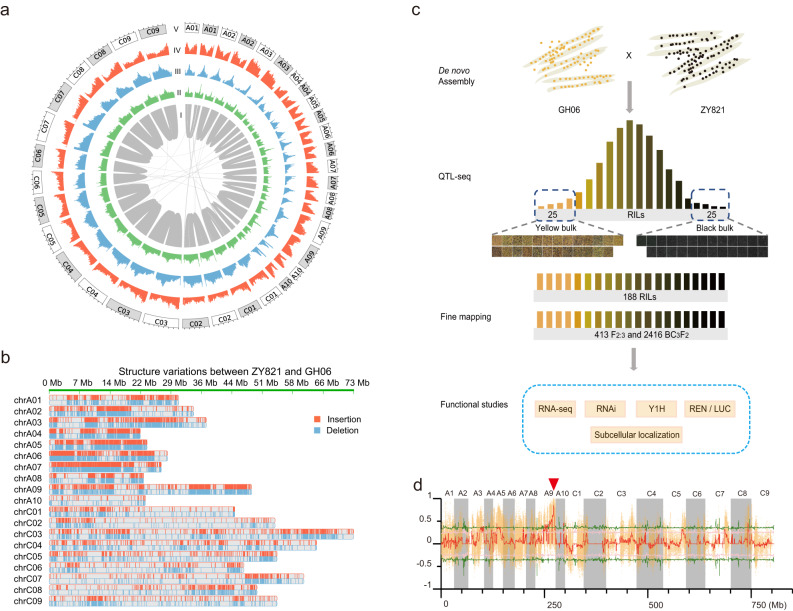
Characteristics of the GH06 and ZY821 genomes, phenotypes of the two parental lines and the two extreme bulks, and the QTL-seq results for the RILs. **a** Circos plot showing the characteristics of the GH06 and ZY821 assemblies. I: Syntenic regions between the GH06 and ZY821 assemblies based on homology searches carried out with MCscan (python version). II: GC contents in non-overlapping 1-Mbp windows. III: Percent coverage of TEs in non-overlapping 1-Mbp windows. IV: Gene densities calculated based on the number of genes in non-overlapping 1-Mbp windows. V: Lengths of pseudo-chromosomes, with white and gray backgrounds representing the pseudo-chromosomes of GH06 and ZY821, respectively. Each ticket above the pseudo-chromosomes is on a scale of 10 Mbp. **b** Structural variation (SV) distribution between the GH06 and ZY821 genomes, with ZY821 serving as the reference sequence. **c** Schematic diagram of the technical roadmap for this study. The yellow- (Y1-Y25) and black-seeded (B1-B25) B. napus lines were used to construct the extreme bulks. Scale bar, 1.0 mm for GH06 and ZY821 and 0.5 mm for Y1–Y25 and B1-B25. **d** QTL-seq applied to the RIL populations identified the seed coat color locus. The permutation test was used for this analysis. Single nucleotide polymorphism (SNP)-index plots showing Δ(SNP-index) (in red) along the 19 chromosomes with statistical confidence intervals under the null hypothesis of no QTLs (green, *P* < 0.01; pink, *P* < 0.05). The red inverted triangle indicates the location of the significant locus.

To study the molecular mechanism of seed color development in *B*. *napus*, we used GH06, a yellow-seeded cultivar, and ZY821, a black-seeded cultivar, as parents to construct a mapping population. We sequenced these two parental genomes using the PacBio Sequel platform, which generated 117.44 Gbp (139×) and 116.99 Gbp (136×) of data from GH06 and ZY821, respectively (Supplementary Data 1). We used the self-corrected PacBio reads for constructing contigs, which were then polished using Illumina paired-end reads (Supplementary Data 2), and produced contig assemblies with N50 of 3.65 Mbp and 3.64 Mbp for GH06 and ZY821, respectively. We then used Dovetail Hi-C data to refine these assemblies (Supplementary Fig. 2a, b, Supplementary Data 2). Approximately 94.04% of the 846.29 Mbp GH06 assembly and 93.34% of the 863.16 Mbp ZY821 assembly were assigned to 19 pseudo-chromosomes (Fig. 1a), with scaffold N50 of 47.79 Mbp and 48.81 Mbp, respectively. The quality of the two assemblies was comparable to that of other *B. napus* reference genomes^28–31^ (Table 1, Supplementary Data 3). The collinearity analyses results showed that the difference in the length of the genome assemblies between GH06, ZY821 and *B. napus* reference genomes was mainly caused by the differences in the assembly of centromere and telomere repeat sequences (Supplementary Figs. 3–8). The pseudo-chromosomes of GH06 and ZY821 showed high collinearity, except for a large inversion on chromosome C09 (Supplementary Fig. 9), where the assembly was supported by Hi-C data (Supplementary Fig. 10). By comparing the two assemblies, we detected 18,505 structure variations (SVs), including 10,208 insertions and 8297 deletions (Supplementary Data 4). Notably, the A sub-genome (with 11,937 SVs) exhibited more variations than the C sub-genome (with 6332 SVs) (Fig. 1b), suggesting that the A sub-genome has undergone more genetic recombination events during *B. napus* evolution. Furthermore, Benchmarking Universal Single-Copy Orthologue (BUSCO) results showed that 99.6% and 99.7% of the 1614 core genes in the OrthoDB Embryophyta database^32^ were detected in the GH06 and ZY821 assemblies (Table 1), respectively. These results confirmed the completeness of these de novo-assembled genomes.

**Table 1:** Comparison of *B. napus* assemblies

Assembly feature	GH06	ZY821		Darmor-*bzh* (v9)
Size of assembly (bp)	846,287,877		863,155,277	1,207,123,300
Contig N50 (bp)	3,648,614		3,639,159	80,133
Scaffold N50 (bp)	47,787,757		48,811,115	55,121,350
Longest scaffold (Mbp)	73,870,175		73,444,947	110,087,657
Anchored pseudo-chromosomes (%)	94.04		93.34	77.32
Number of protein-coding genes	97,639		98,067	102,845
Complete BUSCOs (%)	99.6		99.7	99.7

The GH06 and ZY821 assemblies contained 50.99% and 51.67% repetitive sequences, respectively (Supplementary Data 5). The distribution of the divergence rates for each type of transposable element (TE) was similar for the two genomes (Supplementary Fig. 11). Although the overall abundance of repetitive sequences was similar between the two assemblies, the proportion of Copia-type repeated sequences varied considerably (7.64% vs. 11.57%) (Supplementary Data 6). We annotated 97,639 and 98,067 protein-coding genes with strong evidence in GH06 and ZY821, respectively, by combining ab initio prediction, homologous protein prediction, and transcriptome alignment. Of these protein-coding genes, 95.60% and 96.08%, respectively, were supported by the functional annotation information (Supplementary Data 7, Supplementary Fig. 12). The average lengths of genes, exons, and introns were similar among the GH06, ZY821, and Darmor-*bzh* (v4.1) assemblies. However, the average gene length of the Darmor-*bzh* (v9) assembly was nearly twice that of GH06, ZY821, and eight other *B. napus* genomes published by ref. ^28^. (Supplementary Data 8). This discrepancy may be due to the different sequencing and assembly strategies used to create the Darmor-*bzh* (v9) assembly. In addition, we annotated 5891 and 6251 non-coding RNAs in the GH06 and ZY821 assemblies, respectively, including 330 microRNAs (miRNAs) in each parent, 813 and 679 transfer RNAs (tRNAs), 3558 and 4099 ribosomal RNAs (rRNAs), and 1190 and 1143 small nuclear RNAs (snRNAs), respectively (Supplementary Data 9). These high-quality parental genomes and the comprehensive variations database obtained here form the foundation for subsequent analyses in this study.

### QTL-seq analysis for seed coat color in *B*. *napus*

The seed color differed substantially between the two parental lines, GH06 and ZY821, and also varied considerably for each parent between different growing environments (Supplementary Data 10). Seed color showed a continuous distribution in the RIL population, indicating quantitative inheritance (Supplementary Fig. 13). Analysis of variance (ANOVA) revealed significant variability in seed color among the individual RILs within the population (Supplementary Data 10).

Based on phenotyping data generated for the RIL population, we subjected two extreme pools for seed color (25 black-seeded individuals and 25 yellow-seeded individuals) to the QTL-seq pipeline analysis (Fig. 1c). We used a higher sequencing depth for the individuals in the two extreme pools than for the parents (Supplementary Data 11). After strict filtering (see Methods), the sequencing depths for the black-seeded and yellow-seeded pools were 44.05 × and 40.75 ×, respectively, ensuring that the average sequencing depth of each sample in the pools was more than 1 ×. The clean data from the two extreme pools and the parents were aligned to the ZY821 and Darmor-*bzh* (v4.1) reference genomes using BWA software^31^. The alignment results showed that we obtained a higher mapping rate using the ZY821 reference genome than the Darmor-*bzh* (v4.1) reference genome. The mapping rate of the four samples using ZY821 as the reference sequence was 99.02–99.70%, and that using Darmor-*bzh* (v4.1) as the reference sequence was 98.37–98.70% (Supplementary Data 12). After using a conservative quality filter pipeline (see Methods), we detected 1.39 million genome-wide homozygous single-nucleotide polymorphisms (SNPs) between GH06 and ZY821 (Supplementary Data 13), of which 44.21% were located in intergenic regions, 14.73% in intronic regions, and 16.62% in coding sequences. Among the coding regions, there were 141,588 synonymous and 87,550 non-synonymous SNPs. Moreover, we identified 260,137 genome-wide insertions/deletions (InDels), of which 38.27% were located in intergenic regions, 22.28% in intronic regions, and 2.90% in coding sequences. Among the InDels in coding regions, we identified 1299 frameshift deletions and 1273 frameshift insertions (Supplementary Data 13).

QTL-seq analysis identified 42.06 Mbp of candidate intervals, of which 24.33 Mbp were located on chromosome A09, which had the highest Δ(SNP-index) value (Fig. 1d). Within this chromosome, the window with the highest value was located at 44.19–45.19 Mbp (Table 2). The Δ(SNP-index) values for the intervals on chromosomes A03, A05, and C08 were only slightly higher than the threshold (*P* < 0.01), indicating that these were minor QTLs, which could be involved in seed color (Table 2). The pattern of the QTL-seq results using Darmor-*bzh* (v4.1) as the reference sequence was similar to that referenced against ZY821 (Supplementary Fig. 14). Notably, however, we found additional QTLs on the A09 scaffolds when compared with Darmor-*bzh* (v4.1) (Supplementary Fig. 15), indicating that the major QTL regions on A09 were incomplete in the Darmor-*bzh* (v4.1) assembly. We then aligned the A09 scaffold QTL region (chrA09_random: 1580000–4133004) of Darmor-*bzh* (v4.1) to the Darmor-*bzh* (v9) and ZY821 genomes, respectively. The alignment results showed that 87.50% of the sequences could be mapped to the ZY821 A09 chromosome, and 75.39% to the Darmor-*bzh* (v9) A09 chromosome. Thus, using the parental genome of the RIL population as the reference genome improves the reliability of the results and may facilitate subsequent QTL mining. To narrow down the candidate intervals in the major QTL for seed color on chromosome A09, we used a larger RIL population and two different segregating populations (F_2:3_ and BC_3_F_2_).

**Table 2:** Candidate QTL regions of seed coat color in *B*. *napus*

Chromosome	Region start (Mbp)	Region end (Mbp)	Peak delta SNP-index	^a^Peak difference value	Peak window start (Mbp)	Peak window end (Mbp)
ChrA03	28.99	30.32	0.363	0.002	29.14	30.23
ChrA03	30.76	31.8	0.368	0.003	30.76	31.76
ChrA03	32.57	33.87	0.388	0.031	32.84	33.87
ChrA03	35.84	37.91	0.39	0.037	36.18	37.18
ChrA05	9.08	10.19	0.446	0.087	90.8	10.08
ChrA05	11.86	16.38	0.374	0.008	12.14	13.14
ChrA05	17.26	18.28	0.384	0.024	17.26	18.27
ChrA09	22.78	31	0.444	0.092	25.97	26.98
**ChrA09**	**32.7**	**48.81**	**0.857**	**0.497**	**44.19**	**45.19**
ChrA10	18.86	20.91	0.471	0.103	19.91	20.91
ChrC08	40.89	44.18	0.472	0.112	41.26	42.26

^a^Peak difference value is the difference between Δ(SNP-index) and the threshold value (*P* < 0.01). Bold font, The region where the target gene is located.

### Identification of the candidate gene by QTL analysis and fine mapping

**Fig. 2:**
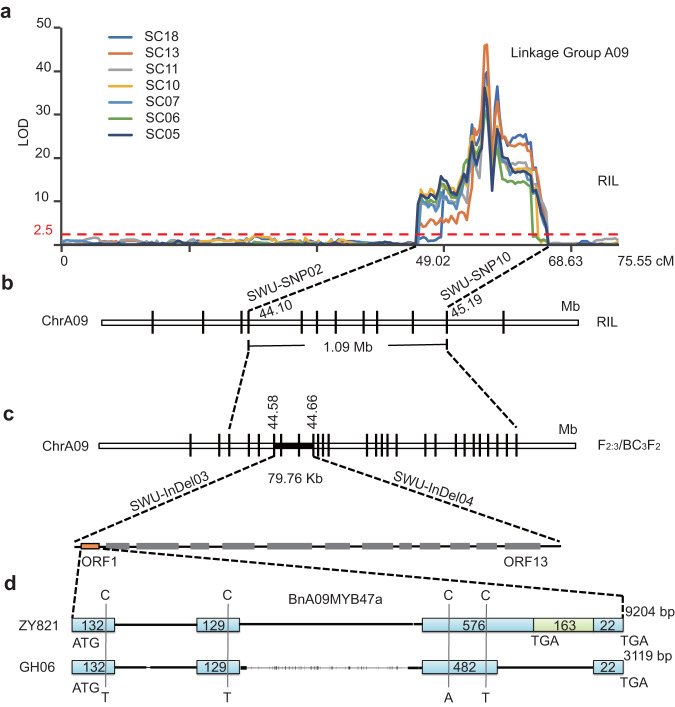
Fine mapping of the seed coat color QTL in *B. napus*. **a** Scanning of the seed coat color QTL (SC) in a *B. napus* RIL population. **b** The seed color locus was detected on chromosome A09 in the RIL population. Positional cloning narrowed the SC locus to a 1.09-Mbp region between SWU-SNP02 and SWU-SNP10. **c** The SC locus was detected on chromosome A09 in the F_2:3_ and BC_3_F_2_ populations. Positional cloning narrowed the SC locus to a 79.76-kbp region between SWU-InDel03 and SWU-InDel04. The orange rectangle indicates the candidate gene. **d** Structure of the *BnA09MYB47a* alleles from black-seeded ZY821 and yellow-seeded GH06 of *B. napus*. The light-blue rectangles represent the exons, and the numbers inside each rectangle represent its size (bp). The green rectangle represents the inserted fragment, and the numbers inside the rectangle represent its size.

The previously reported markers EM11ME20/200 and CB10092/550^17^ were mapped to chromosome A09 of ZY821. To further confirm the location of the candidate QTL on A09, we added 195 SNP markers (using the *Brassica* 60 K Infinium BeadChip Array)^33^, spanning a region of 76.30 cM, with an average distance of 0.39 cM between the two adjacent markers, to the A09 chromosome map. Using composite interval mapping (CIM), we mapped the major QTL interval between markers SWU-SNP02 (SNP21172A09) and SWU-SNP10 (SNP21274A09) (44.10–45.19 Mbp on A09 of ZY821), covering 1.09 Mbp of chromosome A09 within the RIL population, accounting for 45.06–60.35% of the phenotypic variance in seed color (Fig. 2a, b, and Supplementary Data 14 and 15).

To fine-map the major QTL, we generated 413 F_2:3_ individuals and 2,416 BC_3_F_2_ individuals from crosses between the recurrent parents ZY821 and yellow-seed line E393 (Supplementary Fig. 16). A Chi-square goodness-of-fit test indicated that the segregation ratio of seed coat color in the F_2:3_ population deviated from the expected 1:3 ratio (121 yellow vs. 292 black, *χ*^2^ = 1.98, *P* > 0.05). The same was true for the BC_3_F_2_ population, which exhibited a ratio of yellow-seeded lines to black-seeded lines of 549:1867 (*χ*^2^ = 3.45, *P* > 0.05). Using newly developed markers based on InDels and SNPs in the major QTL region of A09 between the parents, GH06 and ZY821, we further delimited the region to between markers SWU-InDel03 and SWU-InDel04 (~79.76 kbp) by PCR identification and homology-based cloning (Fig. 2c and Supplementary Data 16). Based on the ZY821 genome sequence, we annotated 13 candidate genes in this interval, four of which (ZY821040006.1, ZY821040007.1, ZY821040009.1 and ZY821040010.1) showed significant variations (Supplementary Data 17, Supplementary Fig. 17). We further performed sequence alignments for the allelic variation of ZY821040006.1 among different germplasm accessions. Notably, the sequences of ZY821040006.1 were highly similar among the black-seeded accessions but differed substantially from those of the yellow-seeded GH06 and No2127 germplasms^10^ (Supplementary Fig. 18). ZY821040006.1 was annotated as encoding an R2R3-MYB transcription factor, which was the homolog of *Arabidopsis* MYB47 in our phylogenetic analysis (Supplementary Fig. 19, Supplementary Data 18). Therefore, we named the ZY821040006.1 as *BnA09MYB47a*.

### BnA09MYB47a is involved in seed coat pigmentation

**Fig. 3:**
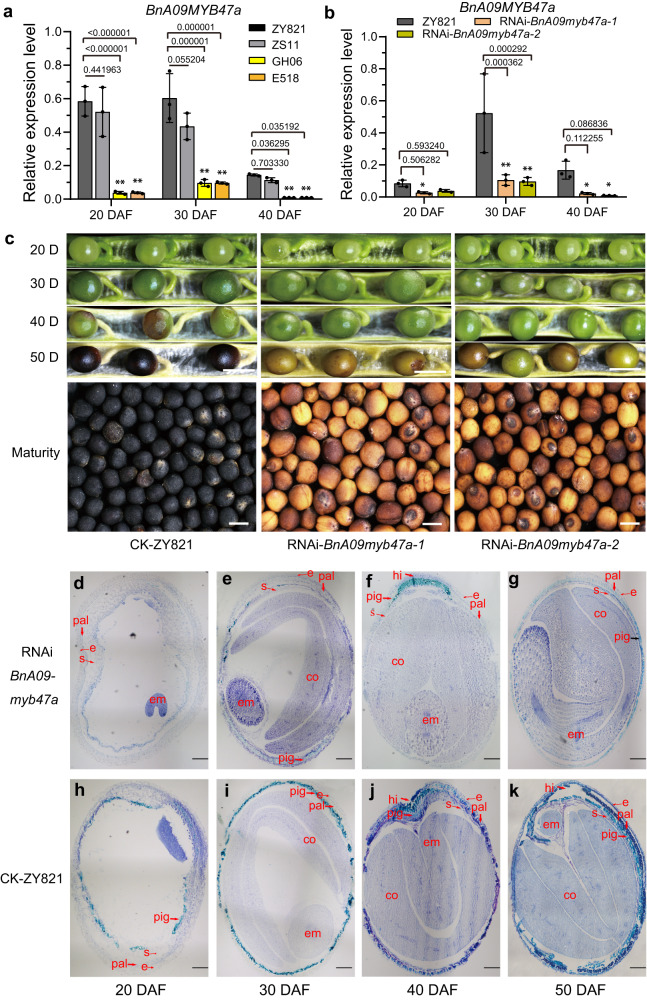
*BnA09MYB47a* is involved in seed coat pigmentation. **a** Relative expression of *BnA09MYB47a* in developing seed coats in different genetic backgrounds. **b** Relative expression of *BnA09MYB47a* in developing seed coats from ZY821 and *RNAi-BnA09myb47a* transgenic plants. In (**a**) and (**b**), seed coat development is divided into three developmental stages (i.e., at 20, 30, and 40 DAF). *BnActin7* (EV116054) was used as the housekeeping gene. *P* values were calculated using multiple *t* tests without adjustments, with comparisons made against the level in ZY821. Values represent the average ± SEM of three biological replicates. **c** Phenotypes of *RNAi-BnA09myb47a* transgenic seeds and control seeds (ZY821) at different developmental stages. DAF, days after flowering. Scale bars, 1 mm, (**d**–**k**) Localization of proanthocyanidins and phenolic compounds (stained with toluidine blue O, in cyan) in the developing seed coat of *RNAi-BnA09myb47a* (**d**–**g**) and ZY821 (**h**–**k**). Assays were performed three times using tissues from three independent experiments. e, epidermis; s, sub-epidermis; pal, palisade layer; pig, pigment layer; hi, hilum; em, embryo; co, cotyledon; DAF, days after flowering. (**d–k**) Scale bars, 20 μm. Source data are provided as a Source Data file.

Cloning and sequencing of the A sub-genome-specific *BnA09MYB47a* from *B*. *napus* revealed an open reading frame (ORF) of 837 bp in the black-seeded ZY821 allele and 765 bp in the yellow-seeded GH06. Sequence alignment between these two ORFs revealed considerable variations, with four SNPs in exons and a large deletion in exon 3 and intron 2 of GH06 (Fig. 2d, Supplementary Fig. 17). In addition, reverse transcription quantitative PCR (RT-qPCR) analysis showed that the expression of *BnA09MYB47a* was significantly lower in developing seeds of yellow-seeded plants (GH06 and E518) than in those of black-seeded plants (ZY821 and ZS9) (Fig. 3a). These results suggest that BnA09MYB47a might be involved in regulating seed coat color.

*AtMYB47* is differentially expressed in *Arabidopsis* seed coats and chalaza, but it is not involved in the yellow-seed trait^34^. To verify whether *BnA09MYB47a* is involved in the seed coat color of *B. napus*, we knocked down *BnA09MYB47a* by RNA interference (RNAi) in black-seeded ZY821 and overexpressed the ZY821 allele (*BnA09MYB47a*^*ZY821*^) driven by the cauliflower mosaic virus *35* *S* promoter (CaMV35S) in yellow-seeded GH06. *BnA09MYB47a* expression was significantly reduced in transgenic RNAi*-BnA09myb47a* lines in the ZY821 background (Fig. 3b). Compared to wild-type ZY821 seeds, the seeds of RNAi*-BnA09myb47a* lines had greatly reduced seed coat color during seed development (Fig. 3c). By contrast, overexpressing *BnA09MYB47a* in GH06 resulted in a darkened seed coat color (Supplementary Fig. 20).

Phenolic compounds, such as flavonols, anthocyanins, and proanthocyanidins (PAs), are the predominant pigments in *B. napus* seed coats^25,35–37^. Toluidine blue O staining, in which cations react with acidic substances such as phenolic compounds and cause them to appear blue^25^, revealed that phenolic compounds were clearly reduced in the RNAi*-BnA09myb47a* lines relative to ZY821 (Fig. 3d–k). We did not observe staining in the hilum of RNAi*-BnA09myb47a* seeds until 40 days after flowering (DAF) (Fig. 3f), whereas these compounds started accumulating in the seed coat at 20 DAF and peaked at 40 DAF in ZY821 (Fig. 3h–k). These results support the hypothesis that BnA09MYB47a functions in seed pigmentation in *B. napus*.

### BnA09MYB47a participates in flavonoid biosynthesis and accumulation

**Fig. 4:**
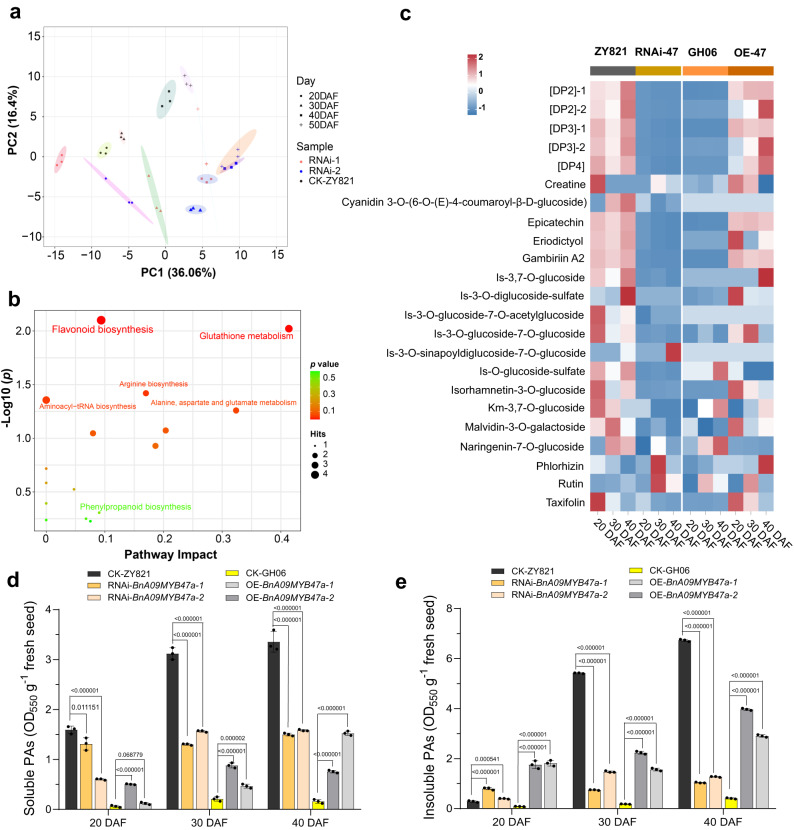
BnA09MYB47a influences flavonoid profile and accumulation. **a** Principal component analysis (PCA) of metabolites in seed coats. Different colored dots represent the ZY821 (black), RNAi-1 (RNAi-*BnA09myb47a-1*, yellow), and RNAi-2 (RNAi-*BnA09myb47a-2*, blue). Different shapes represent the developmental stages of the seed coat. **b** KEGG pathways associated with the biosynthesis of differential metabolites in the seed coat. *P* values by one-tailed hypergeometric test with false discovery rate. **c** Heatmap representing the relative contents of compounds (nmol/g FW) in GH06 and ZY821 (as controls) and transgenic (overexpression [OE] and RNAi) samples, with the ratio expressed in log_2_ scale. RNAi-47, RNAi-*BnA09myb47a*, and OE-47, *BnA09MYB47a*-overexpression. Contents of soluble (**d**) and insoluble (**e**) proanthocyanidins (PAs) in the seed coats of ZY821, GH06, OE-*BnA09MYB47a* and RNAi*-BnA09myb47a* transgenic lines. *P* values were calculated using multiple *t* tests without adjustments, compared to levels in ZY821. Values represent the average ± SEM of three biological replicates. Source data are provided as a Source Data file.

During seed coat pigmentation, the oxidized form of flavonoids and PAs are biosynthesized and accumulated in the seed coats^38–40^. We analyzed the differential metabolites between the seed coats of RNAi*-BnA09myb47a* lines and ZY821 using an ultra-high-pressure liquid chromatography with heated electrospray ionization tandem mass spectrometry (UPLC − HESI − MS/MS) system. Principal Component Analysis (PCA) showed a low variability among biological repeats, meaning that the results were highly reproducible (Fig. 4a). The differentially detected metabolites, including quercetin, isorhamnetin, kaempferol, epicatechin, and their derivatives (Fig. 4c, Supplementary Fig. 21, Supplementary Data 19), were mostly enriched in the flavonoid biosynthesis pathway (Fig. 4b). Among them, [DP2]−1, [DP2]−2, epicatechin, km-3-*O*-glucoside-7-*O*-glucoside, [DP3]−1, [DP3]−2, is-*O*-glucoside-sulfate, and quercetin-3-*O*-glucoside were significantly reduced in the RNAi*-BnA09myb47a* lines compared to ZY821 (Fig. 4c, Supplementary Data 20). Opposite results were observed in the *BnA09MYB47a*^*ZY821*^*-*overexpressing lines in the GH06 background, with the corresponding metabolites being significantly increased compared to GH06 (Fig. 4c, Supplementary Data 21). Furthermore, we measured the PAs levels by directly subjecting the pellet remaining after solvent extraction to oxidative cleavage under hot acidic butanol. PAs levels were significantly increased in the overexpressing lines but significantly reduced in the RNAi lines (Fig. 4d, e). Together, these data indicate that BnA09MYB47a modulates flavonoid biosynthesis and accumulation in *B*. *napus* seed coats.

### BnA09MYB47a positively regulates *BnTT18* in the flavonoid biosynthesis pathway

**Fig. 5:**
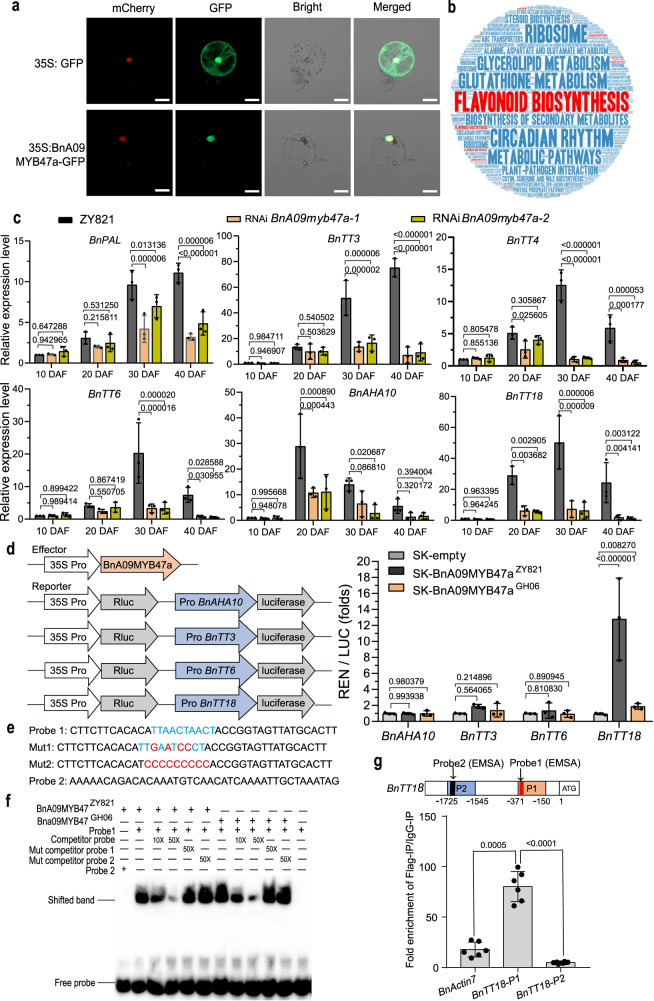
BnA09MYB47a directly regulates flavonoid pathway genes. **a** Subcellular localization of free GFP and BnA09MYB47a-GFP fusion proteins in *B. napu* protoplast*s*. Scale bars, 20 μm. Assays were performed three times using protoplasts from three independent experiments. **b** Word cloud of top 20 significant metabolic pathways (*q*-value ≤ 0.05) for the DEGs between control (ZY821) and RNAi samples. The figure was generated using https://wordart.com/. **c** The relative expression of *BnPAL*, *BnTT3*, *BnTT4*, *BnTT6*, *BnAHA10*, and *BnTT18* in developing seed coats of RNAi transgenic plants compared to those of ZY821, as determined by RT-qPCR. Relative gene expression was normalized to the expression values in ZY821 at 10 days after flowering (DAF). *P* values were calculated using multiple *t* tests without adjustments. Data are presented as average ± SEM of 3 biological repeats. **d** Dual-luciferase reporter assay showing that BnA09MYB47a^ZY821^ rather than BnA09MYB47a^GH06^ regulates *BnTT18* promoter activity. Values are normalized to the level of the blank effector and presented as mean ± SEM from three biological repeats. *P* values were calculated using multiple *t* tests without adjustments. **e** Probes used in the electrophoretic mobility shift assay (EMSA). **f** EMSA was used to assess the binding of BnA09MYB47a^ZY821^ and BnA09MYB47a^GH06^ to the promoter element in *BnTT18*. **g** *BnTT18* is a downstream target of BnA09MYB47a. Upper panel, diagram of the *BnTT18* promoter fragments (P1 and P2) used in chromatin immunoprecipitation-quantitative PCR (ChIP-qPCR). The red and black boxes in P1 and P2, respectively, indicate the detection probes used in the EMSA, as shown in (**e**). Lower panel, BnA09MYB47a-mediated ChIP-qPCR enrichment. Chromatin prepared from transgenic leaves using an anti-Flag antibody (IP) was detected by qPCR with IgG as the control. *BnActin7* and *BnTT18-P2* were used as nonspecific targets. Data are presented as means ± SD (*n* = 6). *P* values were calculated using the two-tailed Student’s *t* test. Source data are provided as a Source Data file.

Flavonoid biosynthesis is tightly regulated by biosynthetic enzymes and various transcription factors (TFs), such as MYB, basic helix-loop-helix (bHLH), and WD40 repeat (WD40) TFs^41–43^. To investigate whether BnA09MYB47a regulates flavonoid biosynthesis, we firstly transiently expressed a construct encoding a BnA09MYB47a-green fluorescent protein (GFP) fusion protein in *B. napus* (Fig. 5a) and *Nicotiana benthamiana* leaves, onion (*Allium cepa*) epidermal cells, and Arabidopsis protoplasts (Supplementary Fig. 22). We exclusively detected the fusion protein in the nucleus, indicating that BnA09MYB47a functions as a transcription factor. Next, we identified 28,757 differentially expressed genes (DEGs) in the seed coats of RNAi*-BnA09myb47a* lines and ZY821 (Supplementary Fig. 23) over the different stages of seed development. Kyoto Encyclopedia of Genes and Genomes (KEGG) analysis showed that these DEGs were enriched in the top 20 significant metabolic pathways (*q*-value ≤ 0.05), and in particular, 74 DEGs (Supplementary Fig. 24) were enriched in the flavonoid biosynthesis pathway (Fig. 5b). Among these 74 DEGs, we validated the expression of six genes (*PAL*, encoding phenylalanine ammonia-lyase; *TT4*, encoding chalcone synthase; *TT3*, encoding dihydroflavonol-4-reductase; *TT6*, encoding flavonol 3’-hydroxylase; *TT18*, encoding leucoanthocyanidin dioxygenases involved in proanthocyanin biosynthesis; and *AUTOINHIBITED H(* + *)-ATPASE ISOFORM 10* (*AHA10*), encoding an H^+^-APTase involved in proanthocyanidin biosynthesis) in the seed coats of RNAi*-BnA09myb47a* lines and ZY821 at 10, 20, 30, and 40 DAF by RT-qPCR (Fig. 5c). As expected, the expression levels of these genes were consistently lower in the seed coats of RNAi*-BnA09myb47a* lines than in those of ZY821 (Fig. 5c).

Next, we compared the transactivation activities of the BnA09MYB47a^ZY821^ and BnA09MYB47a^GH06^ variants using the dual-luciferase reporter assays. BnA09MYB47a^ZY821^ could directly activate the *BnTT18* promoter in vivo, but neither BnaA09MYB47a variant possessed regulatory effects on *BnTT3*, *BnTT6*, or *BnAHA10* promoters (Fig. 5d). We further validated the regulating relationship between BnA09MYB47a and *BnTT18* promoter using a yeast one-hybrid assay (Supplementary Fig. 25a), and proved that *BnTT18* was the target gene of BnA09MYB47a by DNA-affinity purification sequencing (DAP-seq) (Supplementary Fig. 25b, c). Importantly, an electrophoretic mobility shift assay (EMSA) showed that glutathione S-transferase (GST)-MYB47 recombinant protein bound DNA probe 1 containing the core motif (5’-AGTTAGTTA-3’) but not the mutated versions (Mut1 and Mut2) or DNA probe 2 without any core motifs (Fig. 5e, f). Furthermore, we confirmed that BnA09MYB47a^ZY821^ was significantly enriched at the P1 target loci containing DNA probe 1 of *BnTT18* compared to nonspecific targets using chromatin immunoprecipitation-quantitative PCR (ChIP-qPCR) (Fig. 5g). Finally, *BnTT18* was differentially expressed between the three yellow-seeded lines (GH06, L1188, and L1266) and three black-seeded lines (ZY821, ZS11, and L1267), but we did not observe sequence differences in *BnTT18* between these different genetic backgrounds (Supplementary Fig. 26). Collectively, these results indicate that BnA09MYB47a positively regulates flavonoid biosynthesis by directly activating *BnTT18* expression. Our results also indicate that the BnA09MYB47a variant in blacked-seeded ZY821 is functional, whereas the BnA09MYB47a variant in yellow-seeded GH06 is ineffective, resulting in the difference in seed color between these *B. napus* lines.

## Discussion

Although *B. napus* is grown worldwide, especially in Europe, Asia, Australia and North America, yellow-seeded varieties are not extensively cultivated. The yellow-seed trait is sought after in *B. napus* breeding because these varieties tend to have higher oil and protein contents and lower levels of pigmentation than black-seeded varieties in the same genetic background^35–37^. However, naturally occurring *B. napus* genotypes are all black-seeded. The yellow-seed trait is commonly developed by interspecific hybridization^7–10^ and is a quantitative trait influenced by multiple factors, including polygenic control, maternal effects, and environment^3,44–46^.

The first commercial yellow-seeded *B. napus* hybrid was released in 2003, and many elite advanced yellow-seeded breeding lines have since been bred (Supplementary Fig. 1). The yellow-seeded GH06 and black-seeded ZY821 cultivars have been widely used in basic *B. napus* research and breeding programs^3,11,19,24,25,37,47^. To fully exploit their genetic potential, we generated high-quality genome sequences of GH06 and ZY821 by integrating data from PacBio sequencing, Illumina paired-end short-read sequencing, and Hi-C technologies, and revealed extensive genomic and phenotypic variations between these cultivars. The reference genomes of the parental lines and the mapping population generated in this study improve the mapping accuracy, and reduce the errors caused by the genetic distance of the other publicly available genomes, facilitating the detection of the genetic variations uniquely linked to the yellow-seed trait. Notably, we narrowed a major QTL controlling seed color on chromosome A09 based on reference genomes of the parental lines and the mapping population (Figs. 1, 2).

To date, numerous QTLs^19,21,23,48,49^ and candidate genes^25,39,40,50–53^ involved in seed color have been reported in *B. napus*, while the major causal gene(s) have not been identified, and the detailed molecular mechanism by which seed color is determined in *B. napus* remains largely unknown. In previous reports, several key genes encoding regulatory factors of the flavonoid biosynthetic pathways, including *TTG1*, *TT1*, *TT2* and *TT8*, have been identified and found to influence seed coat pigmentation in various *Brassica* species^39,40,54–57^. Indeed, these genes are differentially expressed between black- and yellow-seeded *B. napus*^25,58–60^. In this study, using high-quality genomes, whole-genome resequencing, RNA-seq analysis, and fine mapping, we identified an R2R3-MYB transcription factor, BnA09MYB47a, that controls seed coat color in *B. napus* (Fig. 3, Supplementary Fig. 20). Arabidopsis *MYB47* is mainly expressed in the seed coat during seed development and contributes to seed longevity^34^, but it is not involved in the yellow-seed trait. However, most flavonoid biosynthesis genes are expressed in black-seeded *B. napus* during seed development^25,35,39,40,47,51,53,55,61^, but they are also expressed in the yellow-seeded lines. It is difficult to produce yellow-seeded *B. napus* lines directly from black-seeded lines by inhibiting the expression of individual flavonoid biosynthesis genes. Therefore, we infer that in *B. napus* many flavonoid biosynthesis genes functionally diverged and/or that their regulation was altered following gene duplications during interspecific hybridization. Accordingly, natural variations in BnA09MYB47a resulted in the yellow-seeded trait in *B*. *napus*.

The amount of flavonoids (Supplementary Data 20) and PAs were significantly reduced in RNAi*-BnA09myb47a* lines compared to wild-type ZY821 (Fig. 4), which is in agreement with previous findings that PAs are major constituents of dark seed coats in *Brassica* species^25,36,37,61–64^. Flavonoid biosynthesis genes were also significantly repressed in seed coats during seed development in the RNAi-*BnA09myb47a* lines (Supplementary Fig. 24). Indeed, homologs of flavonoid biosynthesis genes involved in seed color have been characterized in other *Brassica* crops^25,35,39,40,47,51,53,55,61^. Among these, *TT3* and *TT18* were demonstrated to play important roles in anthocyanins and PAs biosynthesis^65,66^. Here, we revealed that BnA09MYB47a^ZY821^ directly activates *BnTT18* expression (Fig. 5), thereby positively regulating flavonoid biosynthesis. In addition, *BnTT18* was differentially expressed between the yellow- and black-seeded *B*. *napus* cultivars, but did not display sequence differences (Supplementary Fig. 26), indicating that its regulation by BnA09MYB47a is responsible for the differences in seed coat pigmentation. However, the transactivation activity of BnA09MYB47a differed significantly between black-seeded ZY821 and yellow-seeded GH06 (Fig. 5d), indicating that the variations in BnA09MYB47a^GH06^ changed its function (Fig. 2d). The R2R3-MYB, bHLH, and WD40 (MBW) ternary complexes governing the flavonoid biosynthesis pathway are well characterized in many species^43,67,68^. Further studies are needed to determine whether MYB47 is involved in the flavonoid biosynthesis pathway by forming MBW complexes.

**Fig. 6:**
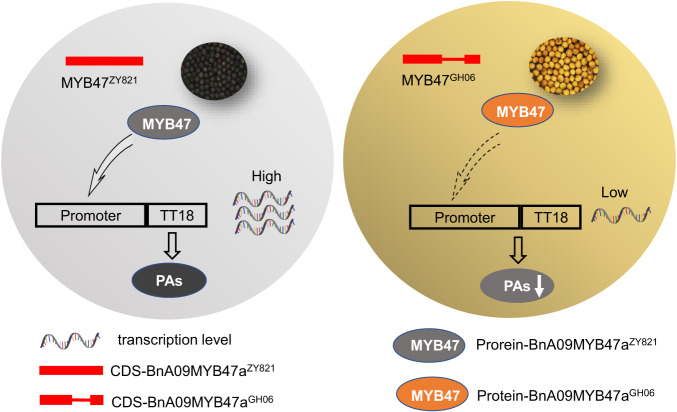
Proposed model of BnA09MYB47a function. BnA09MYB47a^ZY821^ positively regulates flavonoid biosynthesis by directly activating *BnTT18* expression. Whereas the BnA09MYB47a^GH06^ is ineffective, resulting in the difference in seed color between *B. napus* lines.

Overall, our results revealed BnA09MYB47a acted as a critical regulator underlying seed coat pigmentation, and a promising candidate gene for breeding high-quality *B*. *napus*. This study also improves our understanding of the regulation of PAs and anthocyanin biosynthesis in *B*. *napus*, in which the transcription factor BnA09MYB47a has vital roles (Fig. 6).

## Methods

### Plant materials and growth conditions

The male and female rapeseed (*Brassica napus*) parental lines used in this study were yellow-seeded GH06 and black-seeded Zhongyou 821 (ZY821). These plants were characterized previously^25^. The population of 188 recombinant inbred lines (RILs, at the F_2:11_ generation) from a cross between ZY821 and GH06 was grown in a field in Beibei, Chongqing, China in 2005, 2006, 2010, 2011, 2013 and 2018 and their traits were collected for mapping. In addition, the yellow-seeded E518 and black-seeded ZS9 cultivars with different genetic backgrounds were used for RT-qPCR analysis. All experiments were conducted under the same field management conditions.

A segregating population was constructed by self-pollinating the F_1_ plant resulting from the cross between the yellow-seeded line E68 and black-seeded line E85, which were derived from the RIL-1 population^19^ and had generally similar genetic backgrounds. Finally, an F_2:3_ generation consisting of 421 families derived from each F_2_ individual by self-pollination was used to genotype the F_2_ individuals. Another yellow-seeded line, E398, was chosen from among the RILs as the donor parent and was repeatedly backcrossed with ZY821 as the recurrent parent. In the third backcross generation, 2416 BC_3_F_2_ near-isogenic lines (NILs) were chosen based on linked markers, for marker analyses and phenotypic evaluation (Supplementary Fig. 16). The F_2:3_ plants and NILs were grown in a field in Beibei, Chongqing, China from 2009 to 2012. All experiments were conducted under the same field management conditions. Open-pollinated seeds were collected from five randomly chosen plants in each line at maturity for the above analysis.

*Arabidopsis thaliana* (ecotype Columbia-0; Col-0) plants were grown in pots at 22–23 °C in a culture chamber under a 16-h-light/8-h-dark photoperiod with 12,000 lux of supplementary light, and the humidity of the illuminated chamber was approximately 60%.

### PacBio, Illumina and RNA-seq library construction, and sequencing

Two different DNA libraries were constructed and sequenced according to the manufacturers’ instructions: (i) whole-genome sequencing on the PacBio Sequel platform (20-kb library); and (ii) short-read paired-end sequencing (150 bp in length) on the Illumina NovaSeq 6000 platform. To annotate the transcripts of the two genomes (ZY821 and GH06), two strand-specific RNA-seq libraries with an insert size of 350 bp were prepared using the NEBNext® Ultra^TM^ Directional RNA Library Prep Kit for Illumina® (New England Biolabs, USA) and sequenced on the Illumina NovaSeq 6000 platform to generate 150-bp paired-end reads (Frasergen Bioinformatics Co., Ltd., Wuhan, China). Total RNA was extracted from 35 different tissues of ZY821 and three mixed tissues of GH06 and were then sequenced and used to annotate the two genomes.

### Hi-C library construction and sequencing

A Hi-C library was prepared using the Dovetail Hi-C Library preparation kit. Briefly, nuclear chromatin was fixed in plant tissues with formaldehyde and extracted. Fixed chromatin was digested with *Dpn*II, and sticky ends were filled with biotinylated nucleotides and ligated. Then, crosslinks were reversed, and the purified DNA was treated to remove any free biotin from ligated fragments. DNA was then sheared to ~350 bp, and biotinylated fragments were enriched through streptavidin bead pulldown, followed by PCR amplification to generate the library. The library was sequenced on the Illumina NovaSeq platform (Novogene, Tianjin, China).

### Genome assembly

De novo assembly of ZY821 and GH06 was performed using flye (version 2.5) (https://github.com/fenderglass/Flye/)^69^ based on PacBio raw reads with two iterations of polishing. Using the algorithm Arrow (https://github.com/PacificBiosciences/GenomicConsensus), which takes into account all of the underlying data and the raw quality values inherent in SMRT sequencing, the assembly was polished again for the consensus accuracies, and then pillon (v1.22) was used to correct errors introduced into the assembly as a result of errors in the long reads.

To obtain a chromosome-level genome assembly, the Hi-C technique was used to process the temporary assembly. Detailed data processing procedures were as follows: (1) The paired-end Illumina reads were mapped onto the polished temporary genome assembly using HiC-Pro (V2.11.1)^70^ with default parameters to filter the raw Hi-C reads. Self-ligated, non-ligated, and invalid reads (such as PCR amplification, random break, and extreme fragments) were discarded. (2) Juicer (V1.6.2)^71^ and 3D-DNA (version 180114)^72^ were applied to cluster the genomic contig sequences into potential chromosomal groups. (3) JuiceBox (v1.11.8) was employed to validate the contig orientation and to remove ambiguous fragments with the help of manual inspection.

### Annotation of repeats

There are two main types of repeats in the genome: tandem and interspersed. Tandem repeat sequences were identified using Tandem Repeats Finder (TRF, version 4.07)^73^. The interspersed repeat contents of the *B. napus* genome were identified using de novo repeat identification and known repeat searching against existing databases. Two de novo software packages, RepeatModeler (v1.0.8) and LTR_FINDER (v1.0.6)^74^, were used to predict repeat sequences in the genome, RepeatMasker (version 4.0.7) (http://www.repeatmasker.org/) was then used to search the *B. napus* genome against the combined de novo transposable element (TE) library. The homology-based approach to identifying TE repeats in the assembled genome involved applying commonly used databases of known repetitive sequences, RepeatMasker (version 4.0.7) and the Repbase database (version 21). TEs were identified at the DNA level using RepeatMasker and at the protein level RepeatProteinMasker.

### Gene prediction and annotation

Protein-coding region identification and gene prediction were conducted through a combination of homology-based prediction, de novo prediction, and transcriptome-based prediction methods. Proteins from six Brassicaceae plant genomes (*B. juncea*, *B. napus*, *B. nigra*, *B. oleracea*, *B. rapa* and *A. thaliana*) were downloaded from the Brassica Database (http://brassicadb.org/brad/). The candidate homology-based genes were searched by TblastN^75^ with an E-value cutoff of 1e-5. The TblastN results were then processed by Sorting Out Local Alignment Result^76^ to obtain the best hit of each alignment. Subsequently, GeneWise (version 2.4.1)^77^ was used to predict the gene structure of the corresponding genomic regions on each genblasta hit. Three ab initio gene prediction programs, Augustus (version 3.2.1)^78^, GlimmerHMM (version 3.0.4)^79^ and SNAP (version 2006-07-28)^80^, were used to predict coding regions in the repeat-masked genome. Finally, RNA-seq data were mapped to the assembly using hisat2 (version 2.0.1)^81^. Stringtie (version 1.2.2)^82^ and TransDecoder (version 3.0.1) were then used to assemble the transcripts and identify candidate coding regions in gene models. All gene models predicted from the above three approaches were combined by EvidenceModeler (EVM)^83^ into a non-redundant set of gene structures, and the resulting gene models were finally refined with the Program to Assemble Spliced Alignments (v2.3.3)^84^. Protein-coding genes were functionally analyzed using BLASTP (*E*-value cutoff of 1e-05)^85^ against two integrated protein sequence databases: SwissProt and TrEMBL. Protein domains were annotated using InterProScan (V5.30)^86^. The Gene Ontology terms for each gene were extracted with InterProScan. The pathways in which the genes might be involved were assigned by BLAST against the KEGG databases (release 84.0)^87^, with an *E*-value cutoff of 1e-5.

### Annotation of non-coding RNAs

Transfer RNA (tRNA) genes were predicted by tRNAscan-SE (version 1.3.1)^88^. Ribosomal RNA (rRNA) fragments were identified by aligning to Arabidopsis and rice (*Oryza sativa*) template rRNA sequences using BlastN (version 2.2.24) at an *E*-value cutoff of 1e-5. MicroRNA (miRNA) and small nuclear RNA (snRNA) genes were identified by searching against the Rfam database (release 12.0) using INFERNAL (version 1.1.1)^89^.

### Whole-genome sequencing of bulked DNA

The RIL population, comprising 188 individuals, was constructed by crossing GH06 (yellow-seeded) and ZY821 (black-seeded) followed by 11 generations of selfing. Twenty-five lines from each phenotype extreme (yellow seeds and black seeds) were selected for developing two extreme bulks. DNA was extracted from 100 mg of fresh leaves from each line using the DNeasy Plant Mini Kit (QIAGEN Sciences), and then DNA from each line was mixed in an equal ratio. Libraries of 350-bp insert size were constructed and sequenced with 150-bp paired-end reads on the Illumina Xten platform according to the manufacturer’s standard protocols (Novogene, Tianjin, China). The two parental lines, GH06 and ZY821, were also sequenced.

### Sequencing data filtering, alignment, and variation calling of the bulked DNA

Raw reads were filtered by Trimmomatic (version 0.36)^90^ based on their quality score in five steps: (1) removal of adapters; (2) removal of bases from the start or end of a read; if the Phred quality was lower than 3; (3) scanning of the reads with a 4-bp sliding window, removing the window when the average Phred quality per base was below 15; (4) discarding reads with lengths less than 75 bp; and (5) retaining only paired reads. Clean reads were aligned to the ZY821 and Darmor-*bzh* (v4.1) genome sequences^31^ using BWA software (v0.7.12)^91^ with the parameter: ‘mem -t 4 -k 32 –M’. PCR duplicates were removed using SAMtools (v1.3.1)^92^. SNP and InDel calling was performed using the Genome Analysis Toolkit (v3.7-0-gcfedb67)^93^ with the UnifiedGenotyper approach. To remove the potential false-positive variations, SNPs and InDels with QD < 2.0 or FS > 60.0 or MQ < 20.0 or MQRankSum < –12.5 or ReadPosRankSum < –8.0 were filtered out. Gene-based SNP and InDel annotation were performed according to the GH06 genome annotation using the package ANNOVAR (v2013-06-21)^94^. Upstream and downstream regions were defined as a 1-kb region upstream from the transcription start site or downstream from the transcription termination site, respectively.

### Genotyping and SNP-index calculation of the bulked DNA study

To obtain accurate SNP genotyping data, we further filtered the SNPs using the following criteria: (1) the sequencing depth of each bulk and parent at each site was no less than 5; (2) the SNP in each parent was homozygous with different genotypes; and (3) the genotype of ZY821 was consistent with the ZY821 genome sequence.

The SNP-index for each SNP position was calculated for both the bulks according to QTL-seq method^95^. The method applied sliding window analysis to SNP-index plots with 1-Mbp window size and 10-kbp increment. Windows with SNPs < 10 were skipped, and 100,000 replications of the permutation test were employed to determine the 95% and 99% cutoff values. These intervals were plotted for all the genomic regions with variable read depths.

### Quantification of seed coat color

Seeds were harvested from three to five open-pollinated plants per genotype. The seed coat color was quantified using the Near-Infrared Reflectance Analyzer (DS2500, Foss Analytical A/S)^19,96^. Seed coat color was recorded in seven seasons (2005, 2006, 2007, 2010, 2011, 2013, and 2018). Statistical analyses were performed using SPSS 13.0 software.

### Locus identification for seed color

Total genomic DNA was extracted for SNP analysis^24^. Using the *Brassica* 60 K Infinium BeadChip Array, which included 52,157 Infinium Type II SNP loci from the genomic and transcriptomic sequencing data of genetically diverse *Brassica* germplasms (Isobel Parkin, Agriculture and AgriFood Canada), the genotypes of 188 RILs and the parental lines were generated at the National Key Laboratory of Crop Genetic Improvement, National Subcenter of Rapeseed Improvement in Wuhan, Huazhong Agricultural University, 430070 Wuhan, China. Then genetic linkage analyses^24^ were performed using the software packages MSTmap^97^ and JoinMap 4.0^98^. The QTL for seed color was detected using the Windows QTL Cartographer 2.5^99^ following the composite interval mapping method with a LOD threshold of 2.5 (LR ≥ 11.5). The relative contribution of a genetic component was calculated as the proportion of the additive effect and the phenotypic variance explained by that component^99^. The linkage group order and QTLs in the map were processed using Mapchart 2.1^100^. QTL nomenclature followed that of ref. ^101^.

### Fine mapping of the major locus for seed coat color

A BC_3_F_2_ and an F_2:3_ population were used for fine-mapping the seed color QTL, which was based on a rough mapping of the RIL population. To further pinpoint the location of the recombination nearest to the seed coat color locus, bulked segregant analysis was performed to screening markers linked to seed color^102^. Equivalent amounts of DNA from 20 randomly selected yellow-seeded and 20 randomly selected black-seeded individuals were used to construct a yellow and a black bulk from each of the BC_3_F_2_ and F_2:3_ populations. Then, markers were developed based on the differences between parental sequences in the candidate region. These markers were used to determine the genotypes of the recombinants in the BC_3_F_2_ and F_2:3_ populations. The markers are listed in Supplementary Data 22.

### Candidate gene cloning and transgenic analysis

To identify candidate genes, the DNA sequence information of each candidate gene was mapped to the ZY821 reference genome. To identify the candidate genes in the region of the major QTL on chromosome A09, total RNA extracted from developing seeds of GH06 and ZY821 at 20, 30, and 40 days after flowering (DAF) and sequenced on an Illumina NovaSeq 6000 platform (Novogene, Tianjin, China).

The results of the fine-mapping and candidate gene detection were confirmed using two methods. First, the PacBio subreads of GH06 were mapped to the ZY821 reference sequence using minimap2 software, and the structural variations of the candidate genes were manually confirmed with the IGV tool. Second, RT-PCR was used to determine the candidate genes, cloned using the cDNAs originating from ZY821 and GH06, and sequenced to confirm whether there were sequence differences in candidate genes in ZY821 and GH06. All specific primers used in the RT-PCR analysis and cloning are listed in Supplementary Data 22.

Subsequently, the RNA interference (RNAi)-mediated *BnA09MYB47a* knockdown and the *BnA09MYB47a*-overexpressing lines were separately generated by inserting the corresponding constructs into the pFGC5941 and pCAMBIA-2301 vector and then transforming each vector into *Agrobacterium tumefaciens* strain LBA4404, respectively. Plants were transformed using the floral dip method^103^. Primers used in this experiment are listed in Supplementary Data 22.

### Microscopic observation of seed samples

To understand the variation of seed coat color during seed development, siliques were randomly sampled at 20, 30, 40, and 50 DAF and photographed under a stereoscopic light microscope (Olympus SZ61, Münster, Germany).

### Quantification of proanthocyanidins contents of seeds

PAs contents were quantified using the BuOH-HCl method^104^. Three independent batches of seed samples were assessed. Each sample was assayed in triplicate to obtain a mean value. The PAs content is shown as the sample’s absorbance value per unit weight.

### Flavonoid extraction and UPLC − HESI − MS/MS analysis

Seed coats were harvested at 20, 30, and 40 DAF from individual plants of ZY821, RNAi-*BnA09myb47a*, GH06, and *BnA09MYB47a*-overexpressing lines. The raw flavonoids were extracted from the flash-frozen fresh seed coats (100 mg fresh weight), and subjected to UPLC − HESI − MS/MS analysis^25,37^. Flavonoid metabolites were identified by retention times, mass spectrometry, and public information^25,35–37^. The contents of flavonoid metabolites were calculated using the standard curves of available standards: epicatechin, quercetin, kaempferol, and isorhamnetin (Sigma, Aldrich, Shanghai, China). Metabolomics analysis was also performed using Compounds Discoverer 3.0 software (Thermo Fisher Scientific, CA, USA). The Raw MS data have been deposited in MetaboLights under accession number MTBLS6703 (https://www.ebi.ac.uk/metabolights/search). Chemical structure depiction was retrieved from the PubChem database (https://pubchem.ncbi.nlm.nih.gov). All analyses were conducted in triplicate, and the values were represented as means ± SD of three replicates. Statistical significance was based on Tukey’s test, and *P* ≤ 0.05 was considered to be statistically significant.

### Transcriptomic analysis

To investigate the expression of the genes associated with the seed coat color, total RNA was extracted from developing seeds of GH06, ZY821, and the RNAi*-BnA09myb47a* lines at 20, 30, and 40 DAF, and then treated with DNase I (Thermo Fisher Scientific, Wilmington, DE, USA) to remove genomic DNA. RNA integrity was assessed with a BioAnalyzer 2100 (Agilent Technologies, Santa Clara, USA). RNA libraries were prepared using the NEBNext® Ultra^TM^ Directional RNA Library Prep Kit for Illumina® (New England Biolabs, USA) with an insert size of 300 bp for RNA-seq analysis using Illumina NovaSeq 6000 System, which can generate short reads in the 150 -bp paired-end mode^105^.

### Subcellular localization

The complete open reading frame (ORF) of *BnA09MYB47a*^*ZY821*^ (ZY821040006.1) was amplified and inserted into the pNC-Cam1304-SubC (GFP) vector^106^ with green fluorescent protein located at the C terminus of the target protein (primers used are listed in Supplementary Data 22). The recombinant plasmids were transformed into *B. napus* protoplasts, Arabidopsis, onion, and *N. benthamiana*. pNC-Cam1304-SubC (GFP) was employed as the positive control. Green fluorescence was visualized with a laser scanning confocal microscope (LSM8002400301, Carl Zeiss, Germany) at 15 h after transformation.

### Dual-luciferase reporter assay

The 2000-bp promoter sequences upstream of the start codon of *BnAHA10*, *BnTT3*, *BnTT6* and *BnTT18* were individually inserted into the pNC-Green-Luc vector. The *BnA09MYB47a* coding sequences from ZY821 and GH06 were cloned into the pNC-Green-SK vector. pNC-Green-Luc containing the promoter and the pNC-Green-SK-BnA09MYB47a plasmids were mixed at a ratio of 5:1 and transformed into *B. napus* protoplasts. The ratio of Firefly luciferase (LUC) to Renilla luciferase (REN) activity was measured using the Dual-Glo® Luciferase Assay System (Promega) on a luminescence detector (Promega, GloMax 20/20) at 12–16 h after transformation. The ratio of LUC to REN (LUC/REN) activity was then determined^107^.

### Yeast one-hybrid assay

The promoter sequence (1500 bp upstream of the start codon) of *BnTT18* was cloned into the pNC-AbAi vectors as bait. The fusion plasmid linearized by *Bsp*1191 endonuclease was transformed into Y1H Gold competent cells following the manufacturer’s instructions (Weidi, Shanghai, China). The transformed yeast cells were selected on single dropout medium (SD/-Ura) with a gradient concentration of aureobasidin A (AbA) to identify the optimal concentration that could completely suppress the growth of the transformed yeast cells. The *BnA09MYB47a*^*ZY821*^ (ZY821040006.1) coding sequence was cloned into the pGADT7 vector, and then the recombinant vector was transformed into competent cells containing the bait plasmid. The transformed yeast cells were selected on SD/-Leu/-Ura medium containing 200 ng/mL AbA for DNA-protein interaction analysis. The empty pGADT7 vector was transformed into the bait-specific reporter strain as a negative control. The primers used in the experiments are listed in Supplementary Data 22.

### DNA-affinity purification sequencing

Genomic DNA was extracted with the CTAB method and fragmented to 100–200 bp using the Bioruptor Plus instrument (Diagenode, Liege, Belgium). Magnetic beads were used to screen target fragments and then the DNA fragments were ligated to Illumina sequencing adapters with the NEXT-flex Rapid DNA-Seq Kit (BIOO Scientific, Austin, USA). The HaloTag-BnA09MYB47a fusion proteins and HaloTag empty vector (used as a negative control) were translated in vitro using the TnT® coupled reticulocyte lysate system (Promega) for DAP-seq^108^.

### Electrophoretic mobility shift assay (EMSA)

To produce glutathione *S*-transferase (GST)-tagged proteins, *BnA09MYB47a* was amplified and cloned in the pGEX4T-1 vector and expressed in the *Escherichia coli* strain Rosetta. Recombinant GST-BnA09MYB47a protein was purified by GST-tag Purification Resin (Beyotime, Jiangsu, China). GST fusion proteins was detected by anti-GST antibodies (Abcam, ab9085), which was diluted 1000 folds and used for protein-DNA binding. EMSA was performed using the LightShift Chemiluminescent EMSA kit (Thermo Fisher Scientific, Waltham, USA). The recombinant protein was incubated with labeled probes at room temperature for 20 min; the unlabeled probes were used as a competitor to examine the specificity of binding. Protein-probe complexes were separated by electrophoresis on a native 6% acrylamide gel and the DNA was electroblotted onto nitrocellulose membranes and detected by chemiluminescence. Sequences of DNA probes used in EMSA are listed in Supplementary Data 22.

### Chromatin immunoprecipitation (ChIP) assays

The full-length *BnA09MYB47a* coding sequence was amplified and cloned into the pNC-Cam33FC vector, generating a plasmid vector for expressing the *p35S*::*BnA09MYB47a*-*Flag* fusion. The vector was transformed into *B. napus*, and transgenic leaves were used for ChIP-qPCR^109^. Immunoprecipitations was performed with anti-DDDDK-tag mAb-Magnetic Beads (MBL, D153-11) at 4 °C overnight. The precipitated DNA served as a template for qPCR using the KOD SYBR® Green Mix (TOBOYO, OSAKA, Japan). Primer sequences are listed in Supplementary Data 22.

### Reporting summary

Further information on research design is available in the Nature Portfolio Reporting Summary linked to this article.

### Supplementary information

Supplementary Information

Description of Additional Supplementary Files

Supplementary Data 1

Supplementary Data 2

Supplementary Data 3

Supplementary Data 4

Supplementary Data 5

Supplementary Data 6

Supplementary Data 7

Supplementary Data 8

Supplementary Data 9

Supplementary Data 10

Supplementary Data 11

Supplementary Data 12

Supplementary Data 13

Supplementary Data 14

Supplementary Data 15

Supplementary Data 16

Supplementary Data 17

Supplementary Data 18

Supplementary Data 19

Supplementary Data 20

Supplementary Data 21

Supplementary Data 22

Reporting Summary

### Source data

Source Data

## Data Availability

The genome sequence and annotation data for ZY821 and GH06 and their transcriptomic data have been deposited in the NCBI database under BioProject accession PRJNA770894. Source data are provided with this paper.
