# Supplementary material for: Comparative genomic analyses reveal the genetic basis of the yellow-seed trait in Brassica napus
Source: Nat Commun. 2023 Aug 25;14:5194. doi: 10.1038/s41467-023-40838-1 (PMC10457299; doi:10.1038/s41467-023-40838-1)
Supplement: Supplementary file 1 — Supplementary Information [file 41467_2023_40838_MOESM1_ESM.pdf]

Comparative genomic analyses reveal the genetic basis of the yellow-seed trait in *Brassica napus*

Qu *et al.*

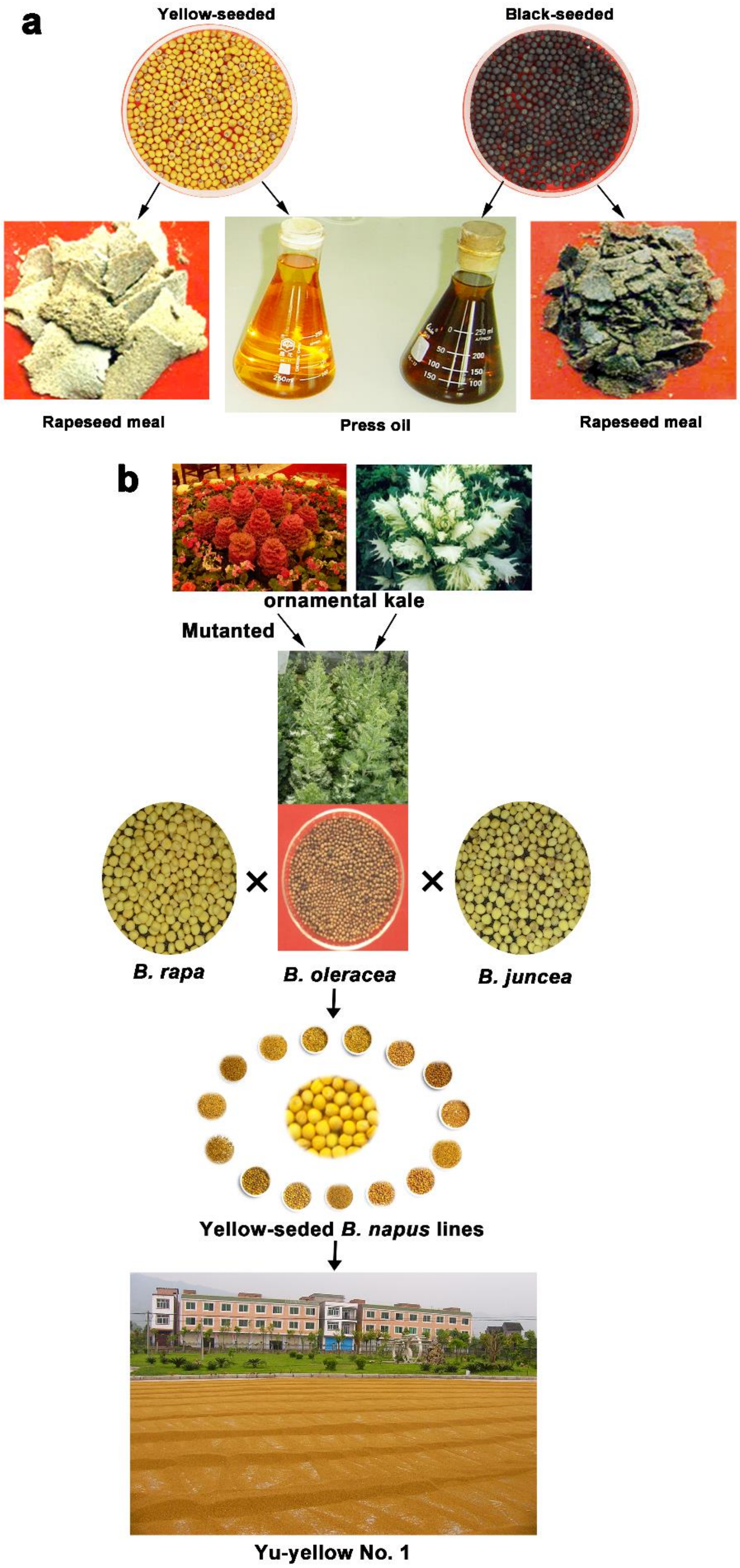

Supplementary Fig. 1. Characterization and origin of yellow-seeded *B. napus*. a, Advantages of the yellow-seeded trait in *B. napus*; **b**, Development of yellow-seeded *B. napus* and Yu-yellow No. 1.

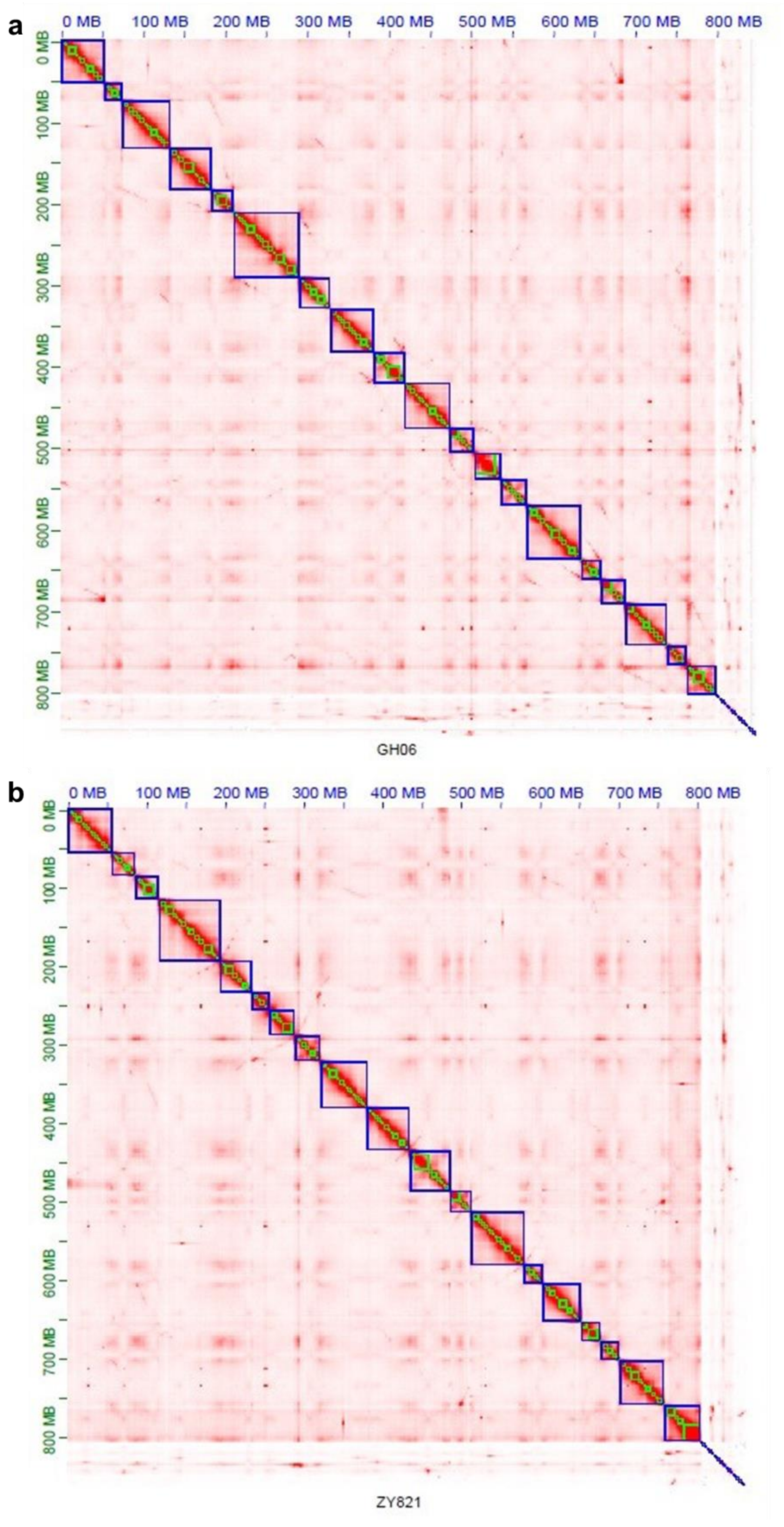

Supplementary Fig. 2. Hi-C contact heatmap. Genome-wide analysis of chromatin interactions in the GH06 genome (**a**) and the ZY821 genome (**b**).

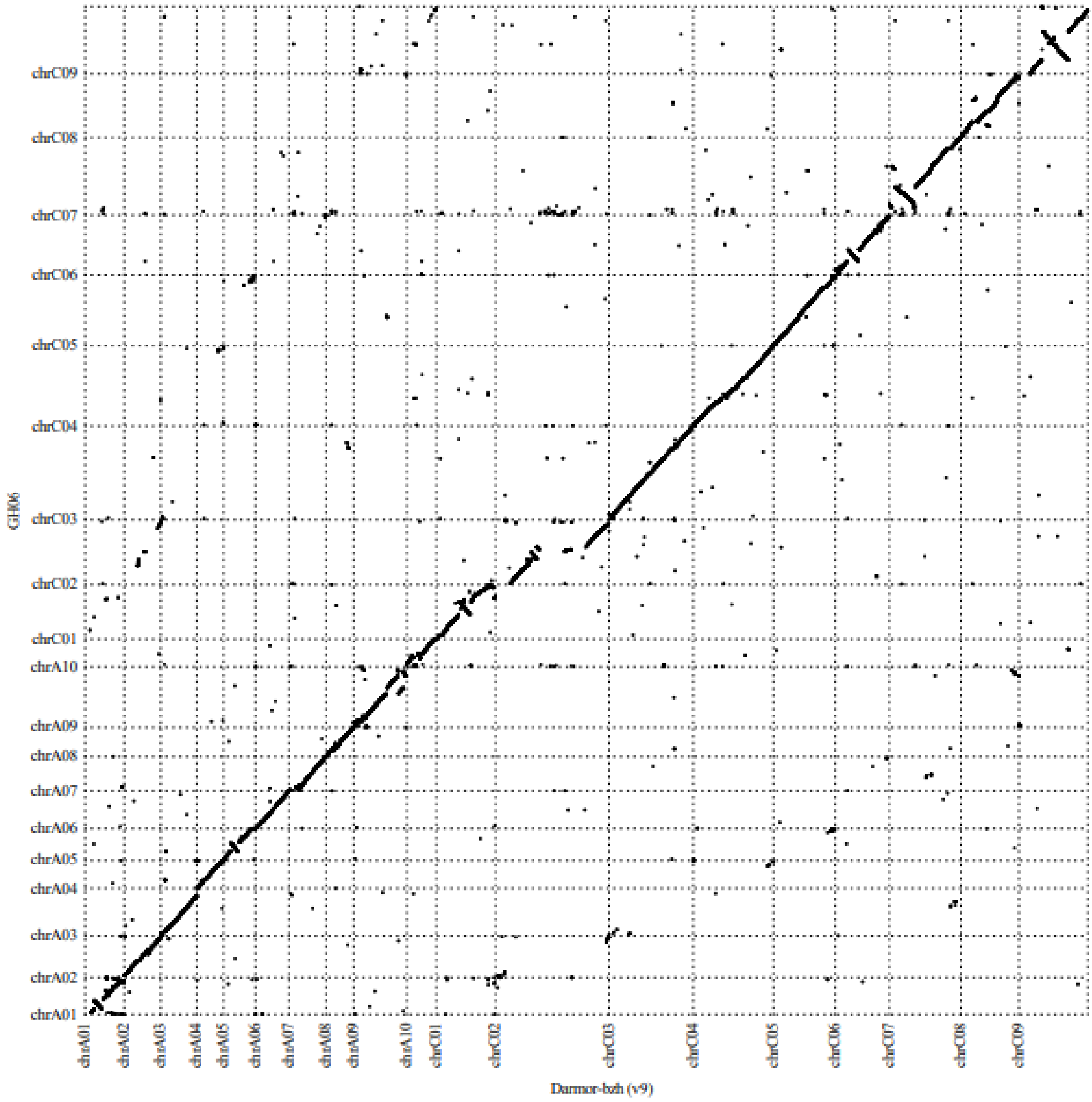

Supplementary Fig. 3. Synteny between the GH06 and Darmor-*bzh* (v9) assemblies. The pseudo-chromosomes of GH06 were aligned to the Darmor-*bzh* (v9) assembly by MUMmer (version 3.23) with default parameters and the genomic alignment results were extracted with the delta-filter -1 -l 10000 parameters.

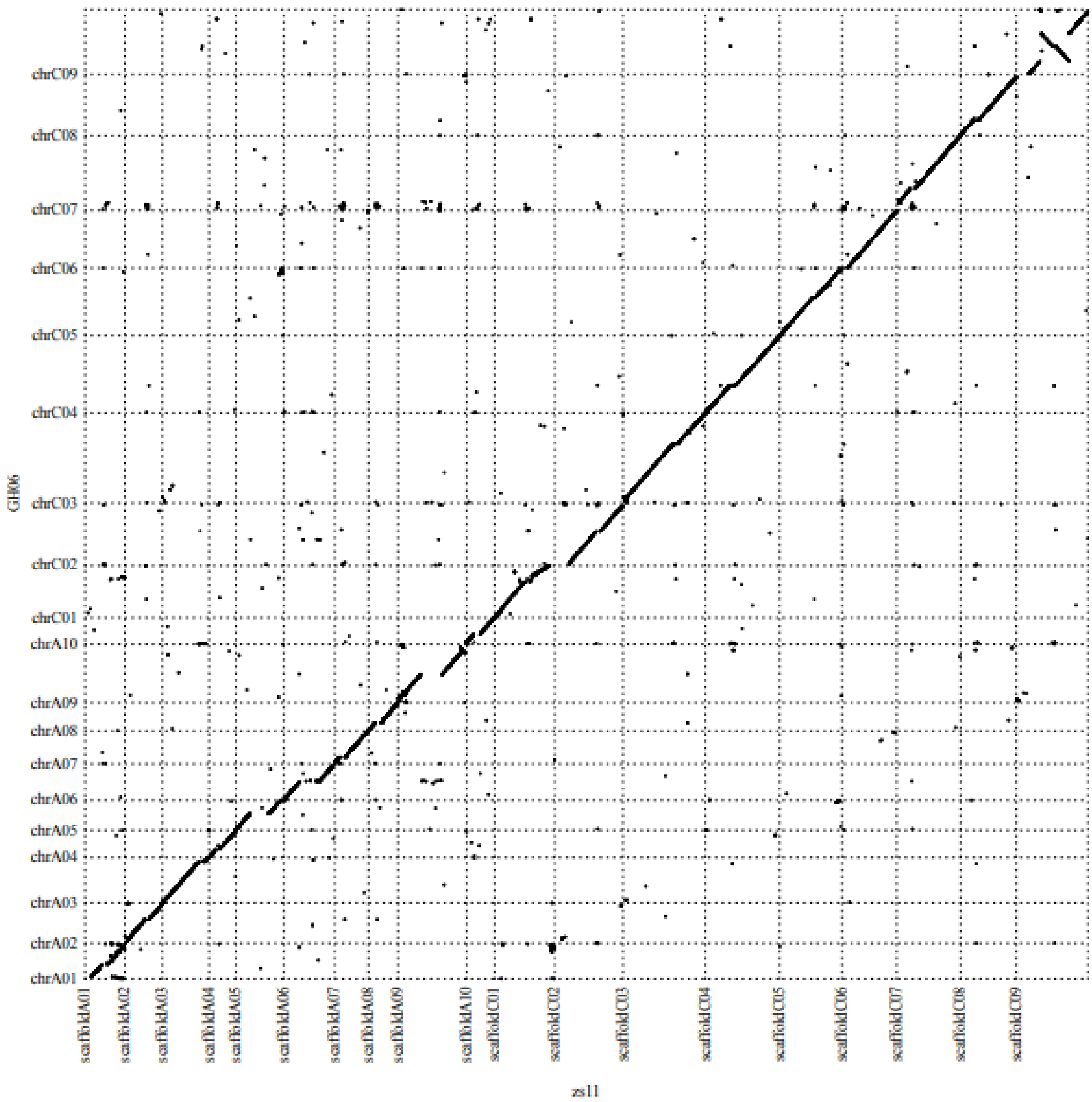

Supplementary Fig. 4. Synteny between the GH06 and ZS11 assemblies. The pseudo-chromosomes of GH06 were aligned to the ZS11 assembly by MUMmer (version 3.23) with default parameters and the genomic alignment results were extracted with the delta-filter -1 -l 10000 parameters.

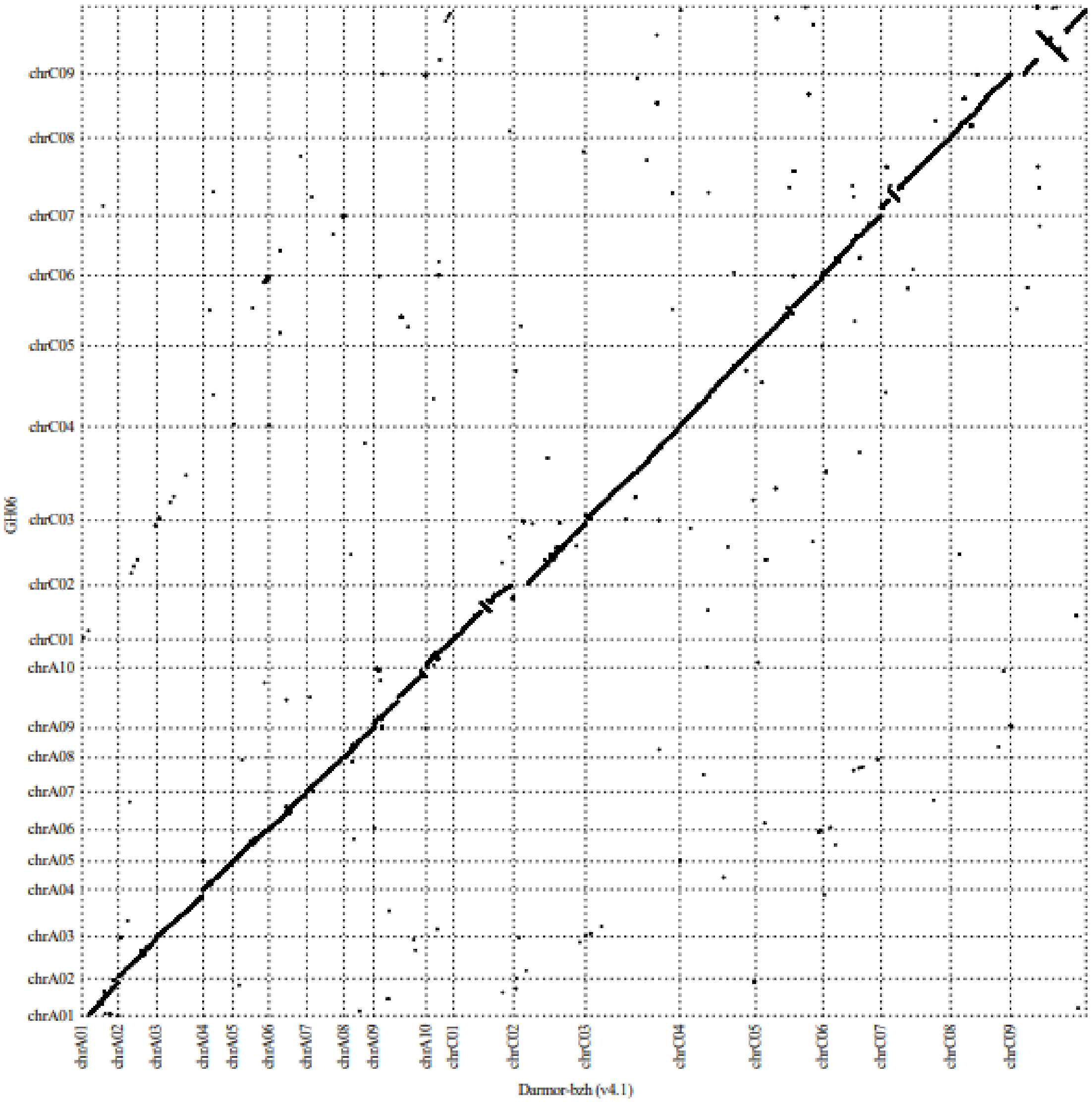

Supplementary Fig. 5. Synteny between the GH06 and Darmor-*bzh* (v4.1) assemblies. The pseudo-chromosomes of GH06 were aligned to the Darmor-*bzh* (v4.1) assembly by MUMmer (version 3.23) with default parameters and the genomic alignment results were extracted with the delta-filter -1 -l 10000 parameters.

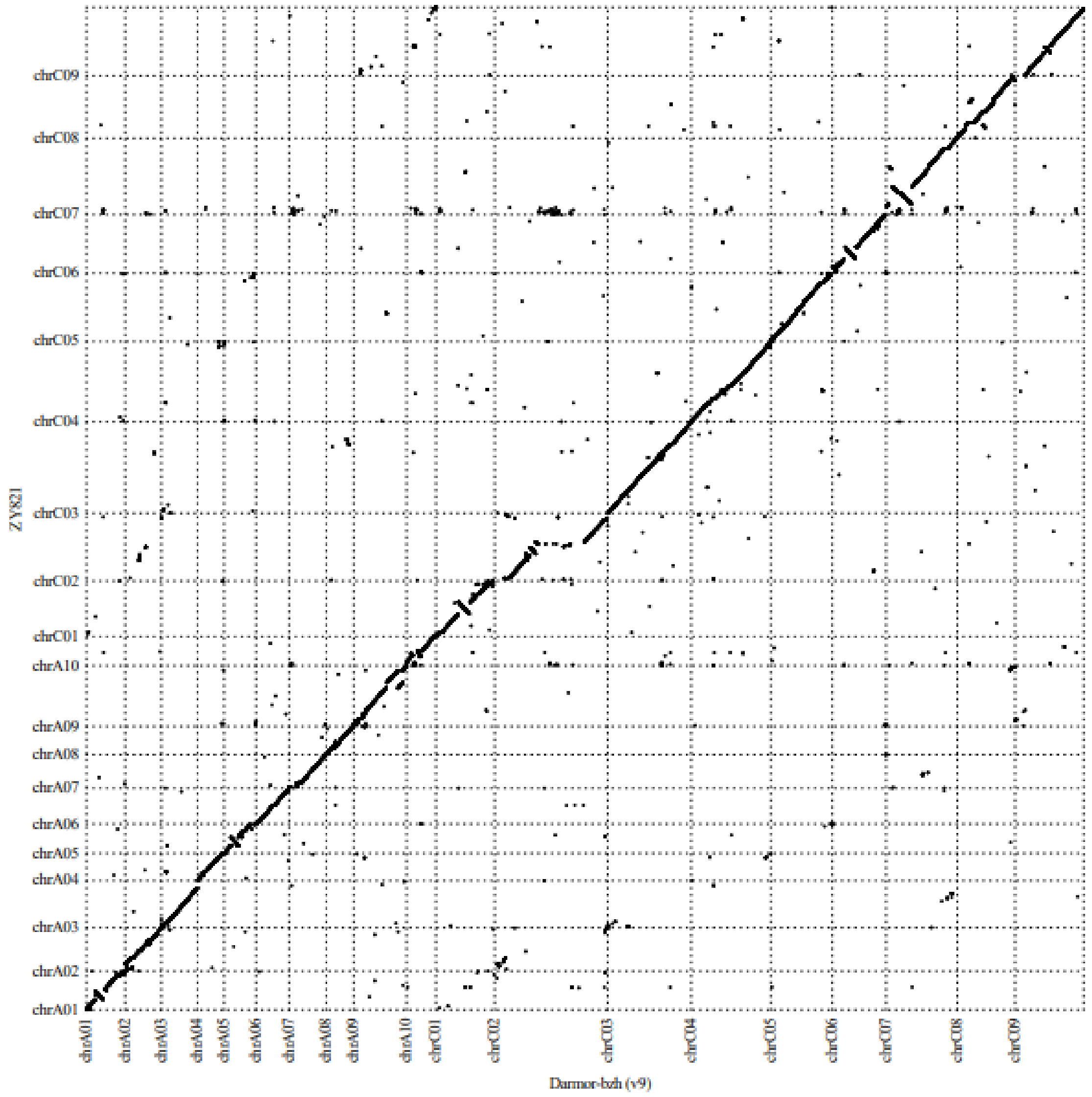

Supplementary Fig. 6. Synteny between the ZY821 and Darmor-*bzh* (v9) assemblies. The pseudo-chromosomes of ZY821 were aligned to the Darmor-*bzh* (v9) assembly by MUMmer (version 3.23) with default parameters and the genomic alignment results were extracted with the delta-filter -1 -l 10000 parameters.

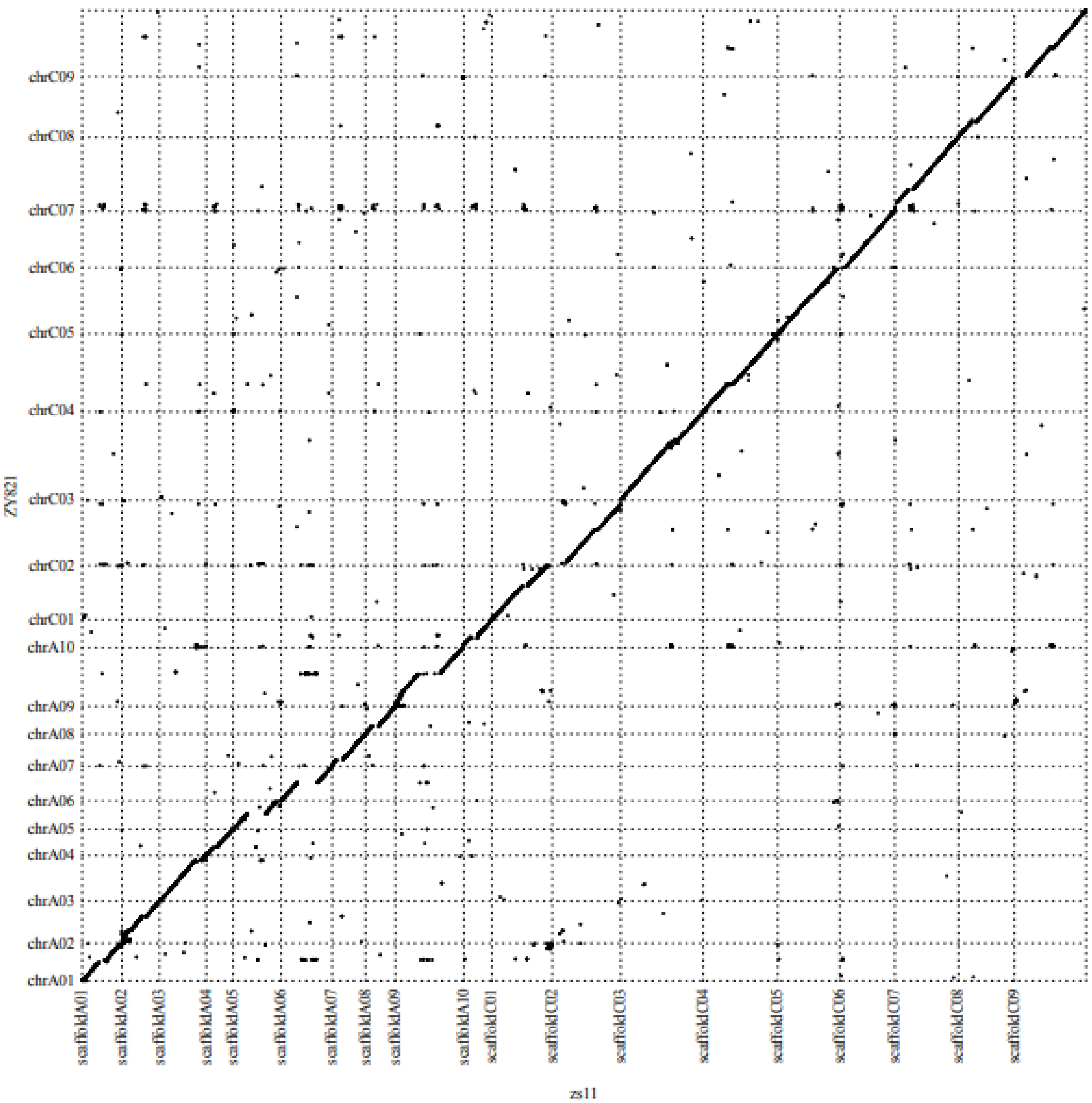

Supplementary Fig. 7. Synteny between the ZY821 and ZS11 assemblies. The pseudo-chromosomes of ZY821 were aligned to the ZS11 assembly by MUMmer (version 3.23) with default parameters and the genomic alignment results were extracted with the delta-filter -1 -l 10000 parameters.

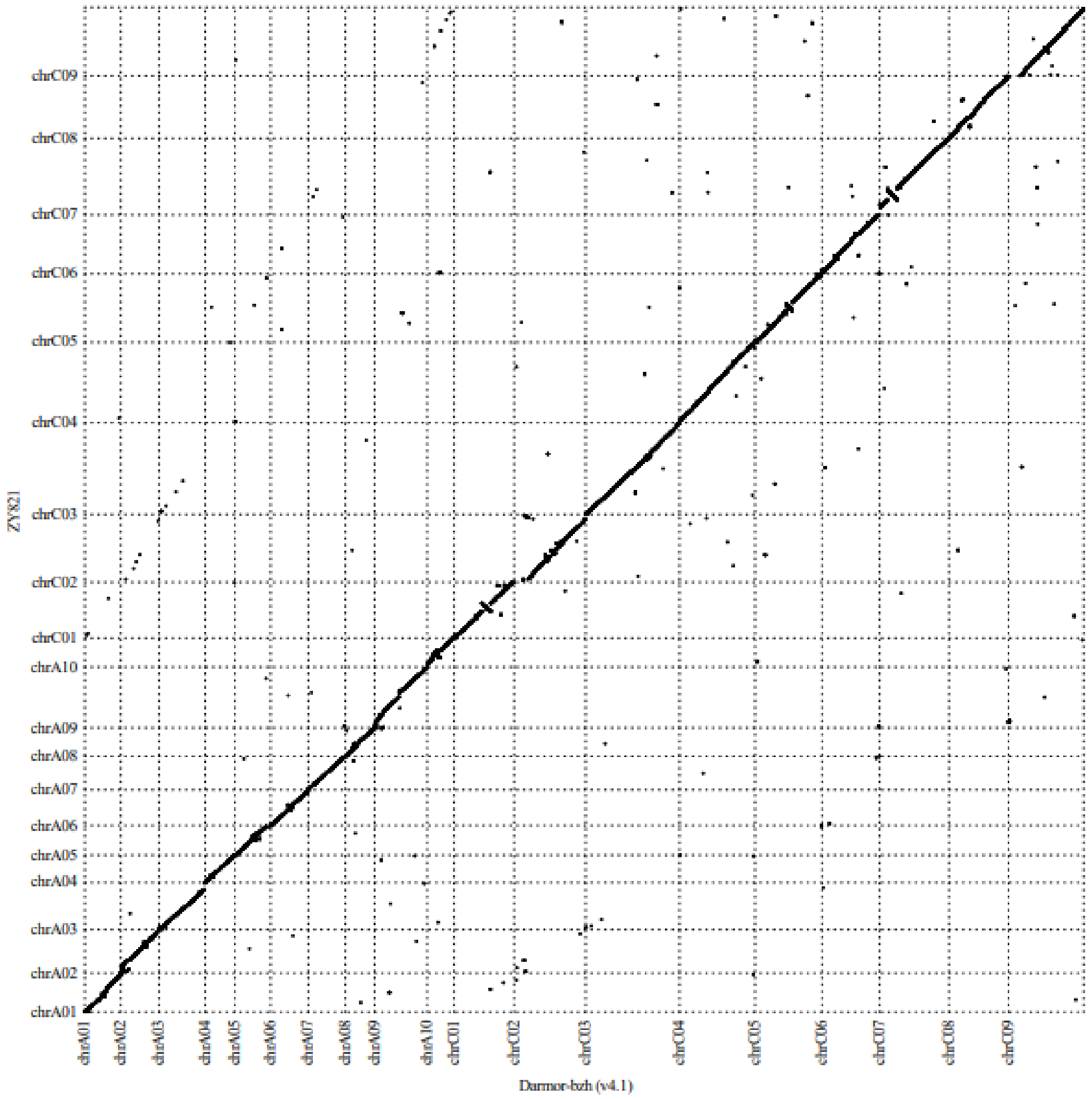

Supplementary Fig. 8. Synteny between the ZY821 and Darmor-*bzh* (v4.1) assemblies. The pseudo-chromosomes of ZY821 were aligned to the Darmor-*bzh* (v4.1) assembly by MUMmer (version 3.23) with default parameters and the genomic alignment results were extracted with the delta-filter -1 -l 10000 parameters.

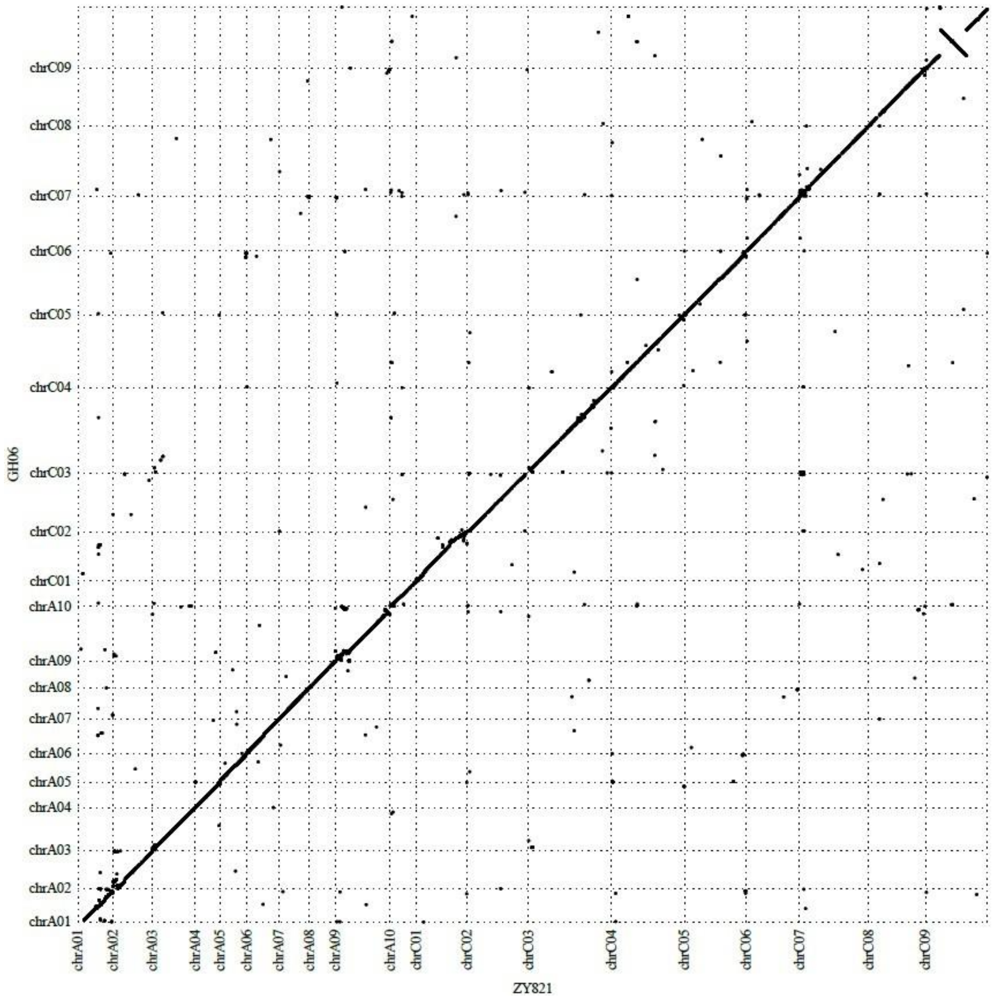

Supplementary Fig. 9. Synteny between the GH06 and ZY821 assemblies. The pseudo-chromosomes of GH06 were aligned to the ZY821 assembly by MUMmer (version 3.23) with default parameters and the genomic alignment results were extracted with the delta-filter -1 -l 30000 parameters.

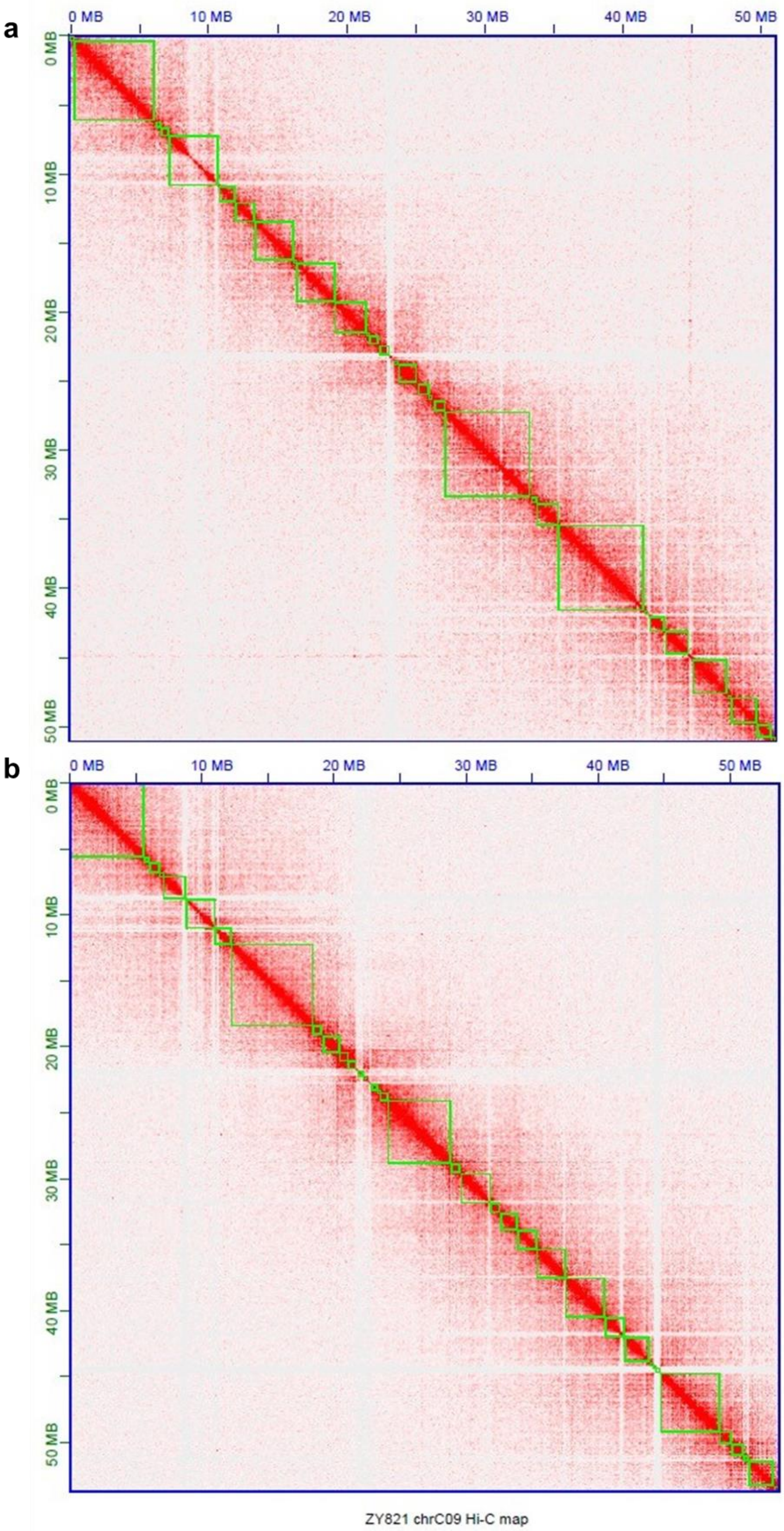

Supplementary Fig. 10. Hi-C contact heatmap of chrC09. a, Hi-C contact heatmap of chrC09 in the GH06 genome. b, Hi-C contact heatmap of chrC09 in the ZY821 genome**.**

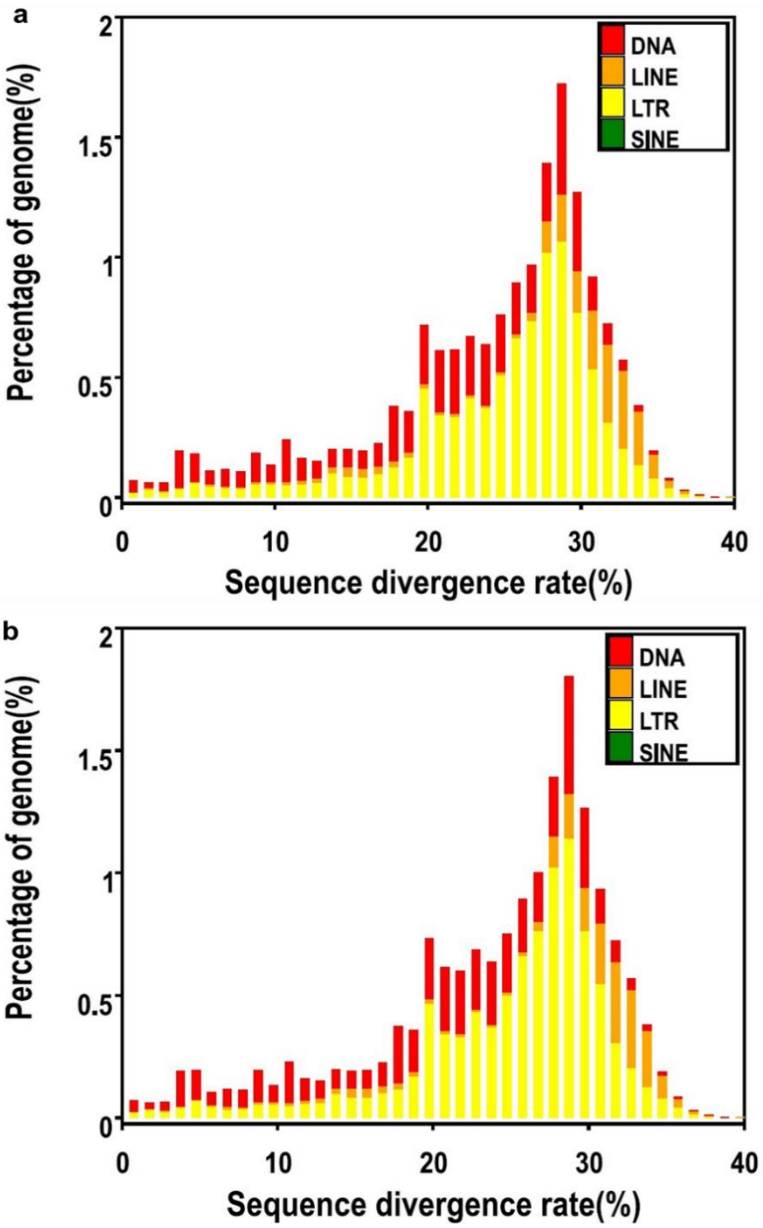

Supplementary Fig. 11. Distribution of the divergence rate of each type of transposable element. a, Distribution of the divergence rate of each type of transposable element in GH06. **b**, Distribution of the divergence rate of each type of transposable element in ZY821. The divergence rate was calculated between the identified TE in the genome by a homology-based method and the consensus sequence in Repbase.

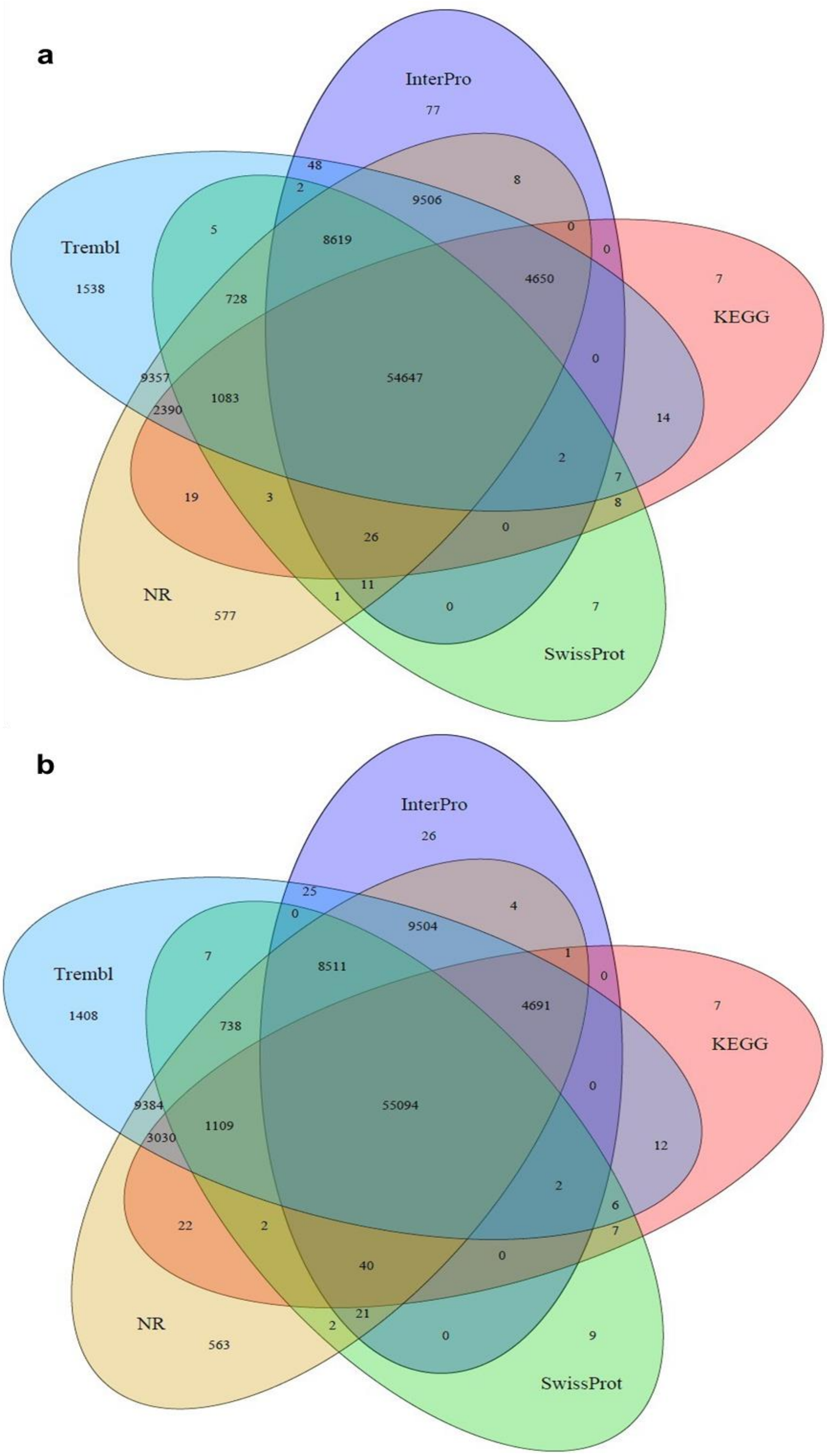

Supplementary Fig. 12. Venn diagrams showing shared genes among different annotated datasets. a, Venn diagrams showing genes shared among different annotated datasets in GH06. **b**, Venn diagrams showing genes shared among different annotated datasets in ZY821.

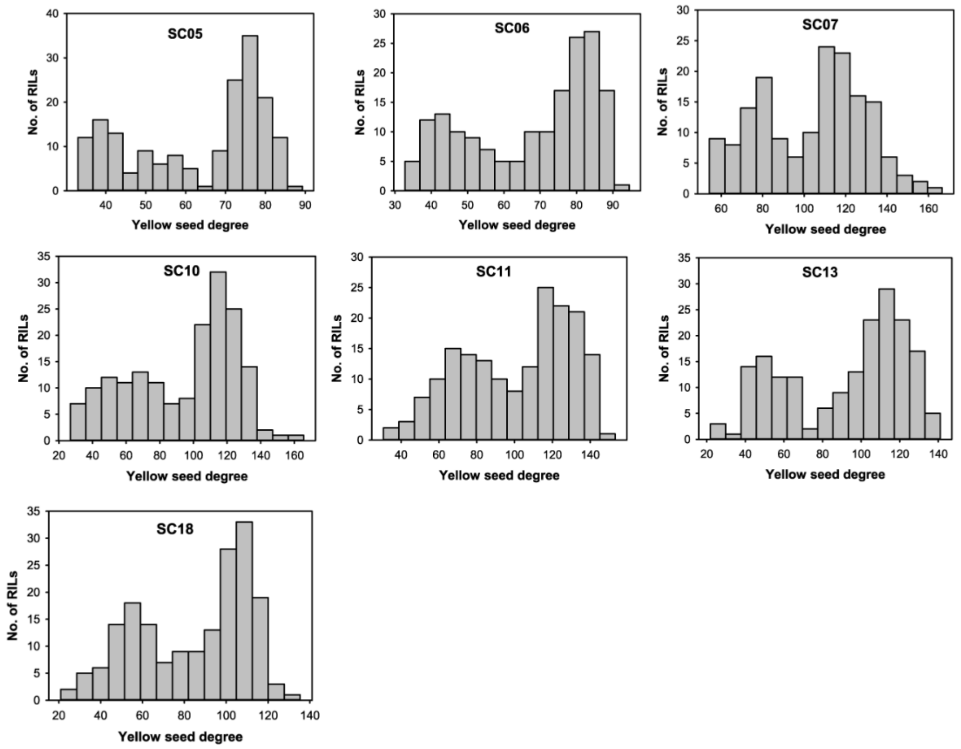

Supplementary Fig. 13. Frequency distribution of seed coat color in recombinant inbred lines. Abscissa: phenotypic value of the traits, Ordinate: number of lines. The name of each line is given in the graph and the culture conditions are indicated by SC05, SC06, SC07, SC10, SC11, SC13, and SC18, respectively. SC means seed coat color in different environments.

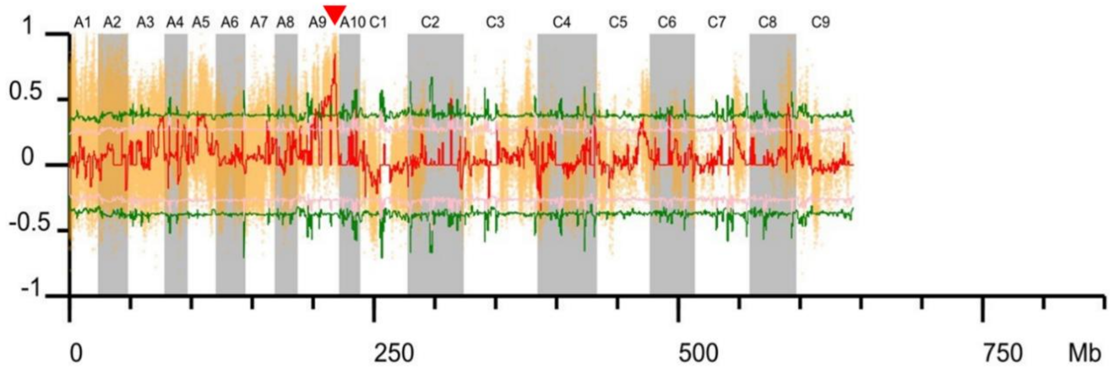

Supplementary Fig. 14. QTL-seq applied to the RIL population identified the seed coat color trait on the Darmor-*bzh* (v4.1) reference genome. Single nucleotide polymorphism (SNP)-index plot showing Δ(SNP-index) (in red) along 19 chromosomes with statistical confidence intervals, under the null hypothesis of no QTLs (green, P <0.01; pink, P <0.05). The red inverted triangle indicates the location of the significant locus.

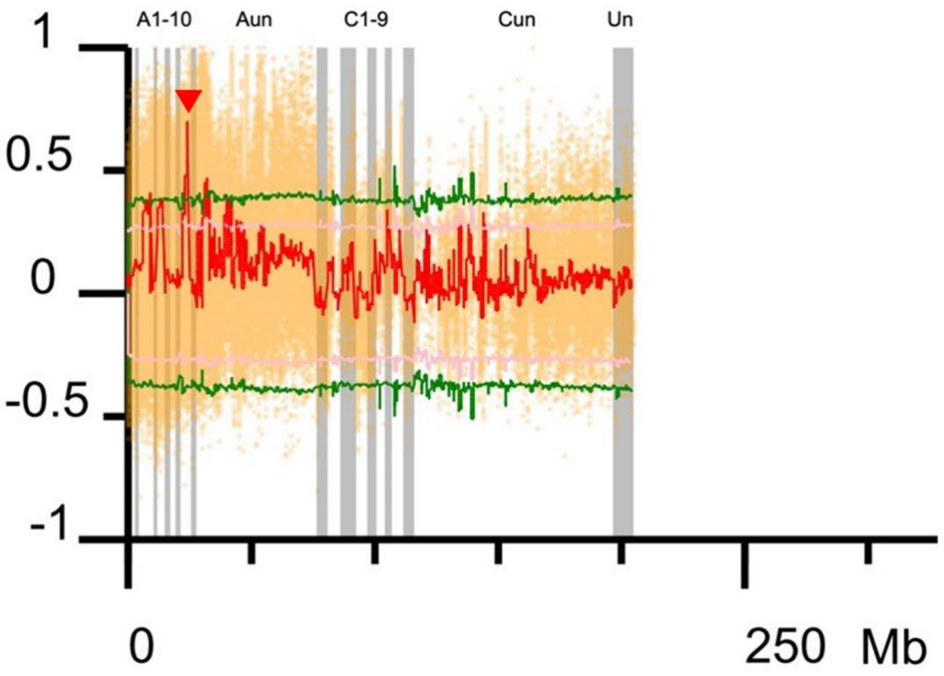

Supplementary Fig. 15. QTL-seq applied to the RIL population identified the seed coat color trait on the Darmor-*bzh* (v4.1) reference genome. Single nucleotide polymorphism (SNP)-index plot showing Δ(SNP-index) (in red) along 19 unanchored A and C chromosomes and scaffolds with statistical confidence intervals under the null hypothesis of no QTLs (green, P <0.01; pink, P <0.05). The red inverted triangle indicates the location of the significant locus.

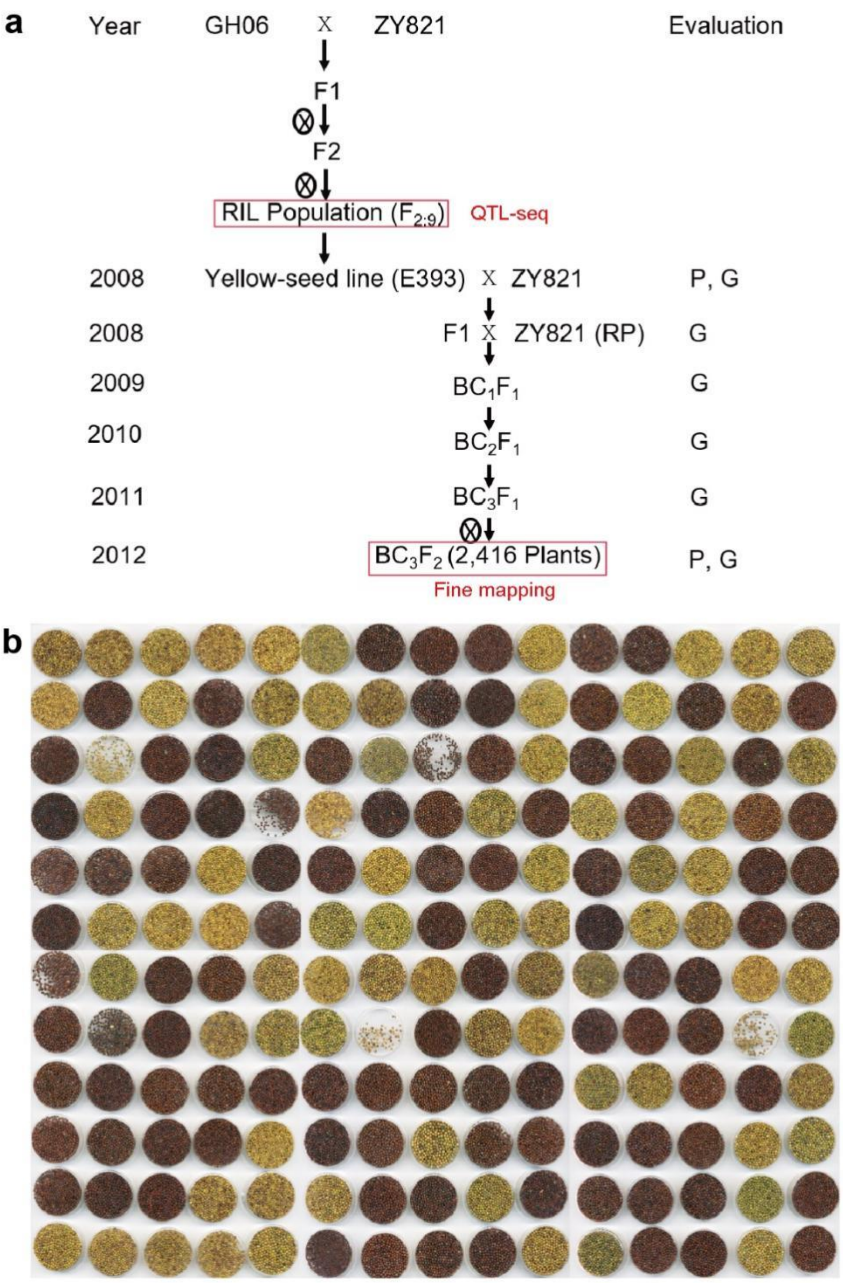

Supplementary Fig. 16. Procedure used to develop QTL-NILs and seed phenotype of BC_3F_2 individuals. a, The procedure used to develop QTL-NILs. P, phenotyping; G, genotyping; X, crosses; Encircled X (⊗), self-pollination; BC, back-crossed. b, Phenotype of BC_3F_2 seeds.

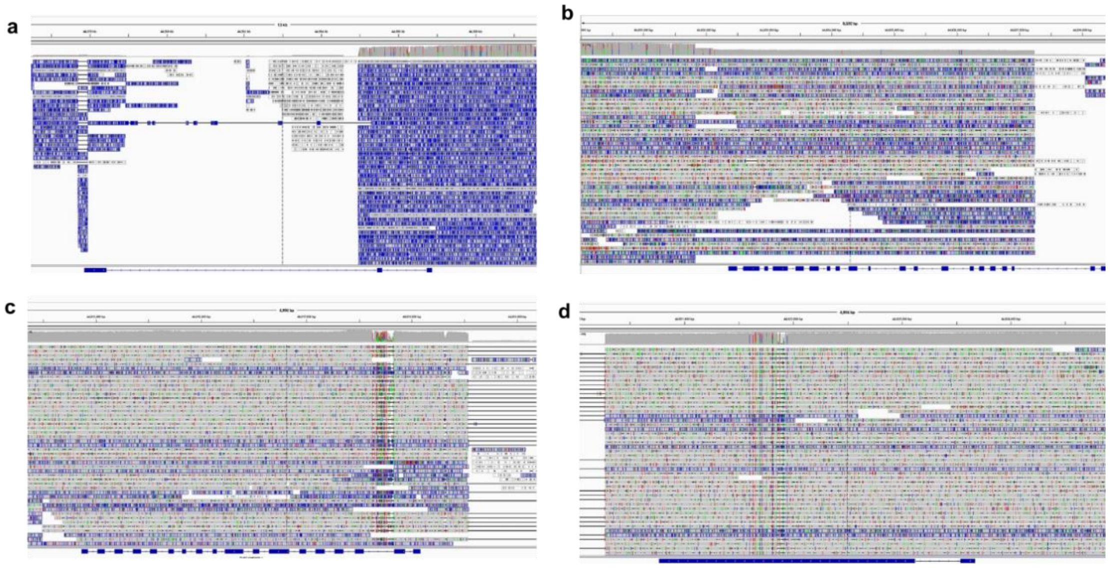

Supplementary Fig. 17. Genes with significant internal variations in the QTL interval regions. a, ZY821040006.1. **b**, ZY821040007.1. **c**, ZY821040009.1. **d**, ZY821040010.1. The PacBio subreads of GH06 were mapped to the ZY821 reference sequence with Minimap2 software, and the structure variations were manually confirmed with the IGV tool.

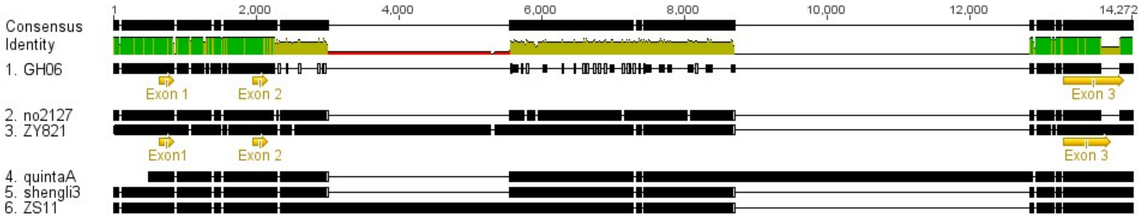

Supplementary Fig. 18. Multiple sequence alignment analysis of *BnA09MYB47a* (ZY821040006.1) from different *B. napus* cultivars. Yellow-seeded lines (GH06 and no2127); Black-seeded lines (ZY821, quintaA, shengli3 and ZS11). The sequence information was downloaded from http://yanglab.hzau.edu.cn/BnTIR.

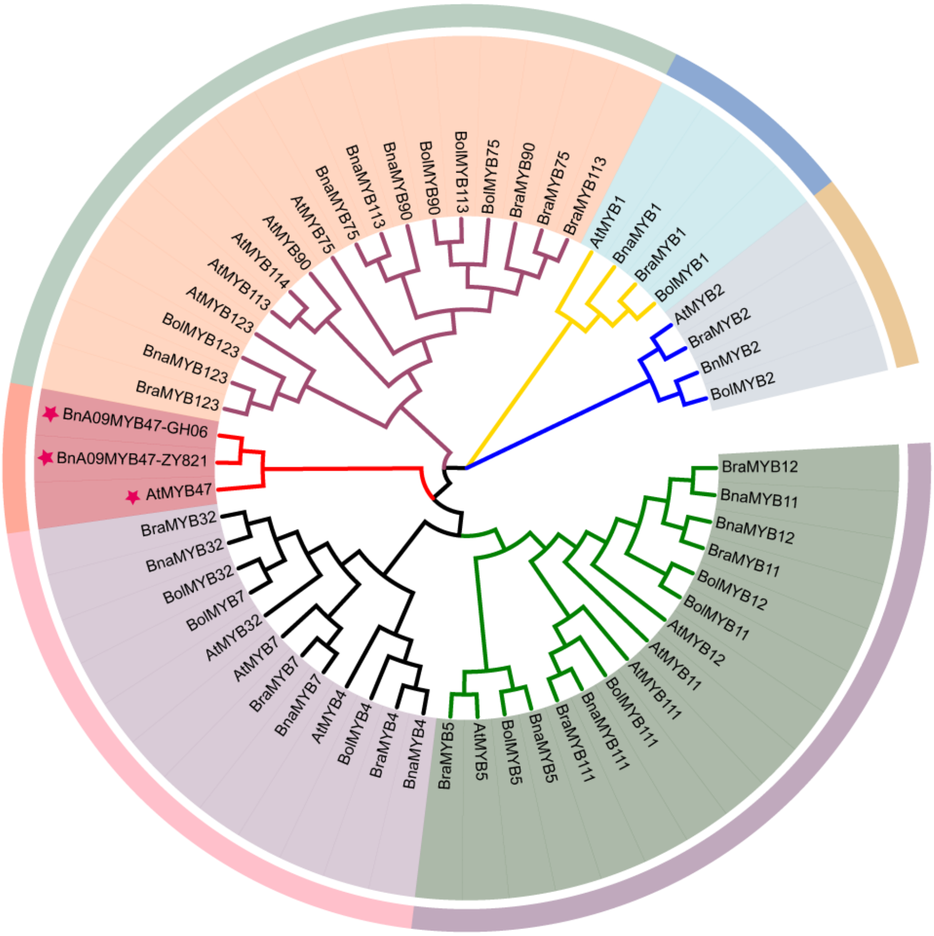

Supplementary Fig. 19. Phylogenetic analysis of the amino acid sequences of BnA09MYB47a with other R2R3-MYB transcription factors from various plant species. The unrooted Maximum Likelihood phylogenetic tree was constructed using MEGA7.0 and visualized using Figure Tree v1.4.2. Organism name and gene accession numbers are shown in Supplementary Table 18.

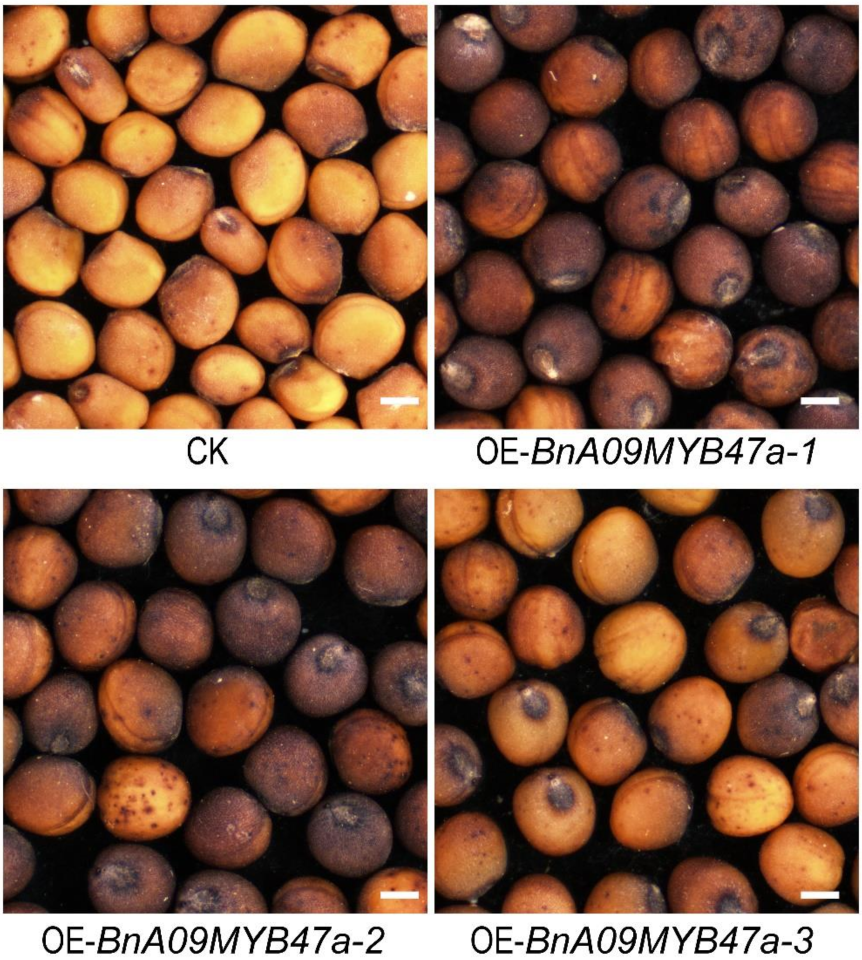

Supplementary Fig. 20. Phenotypes of seeds *BnA09MYB47a*^{ZY821} overexpressing and control seeds (GH06). Scale bars, 1 mm.

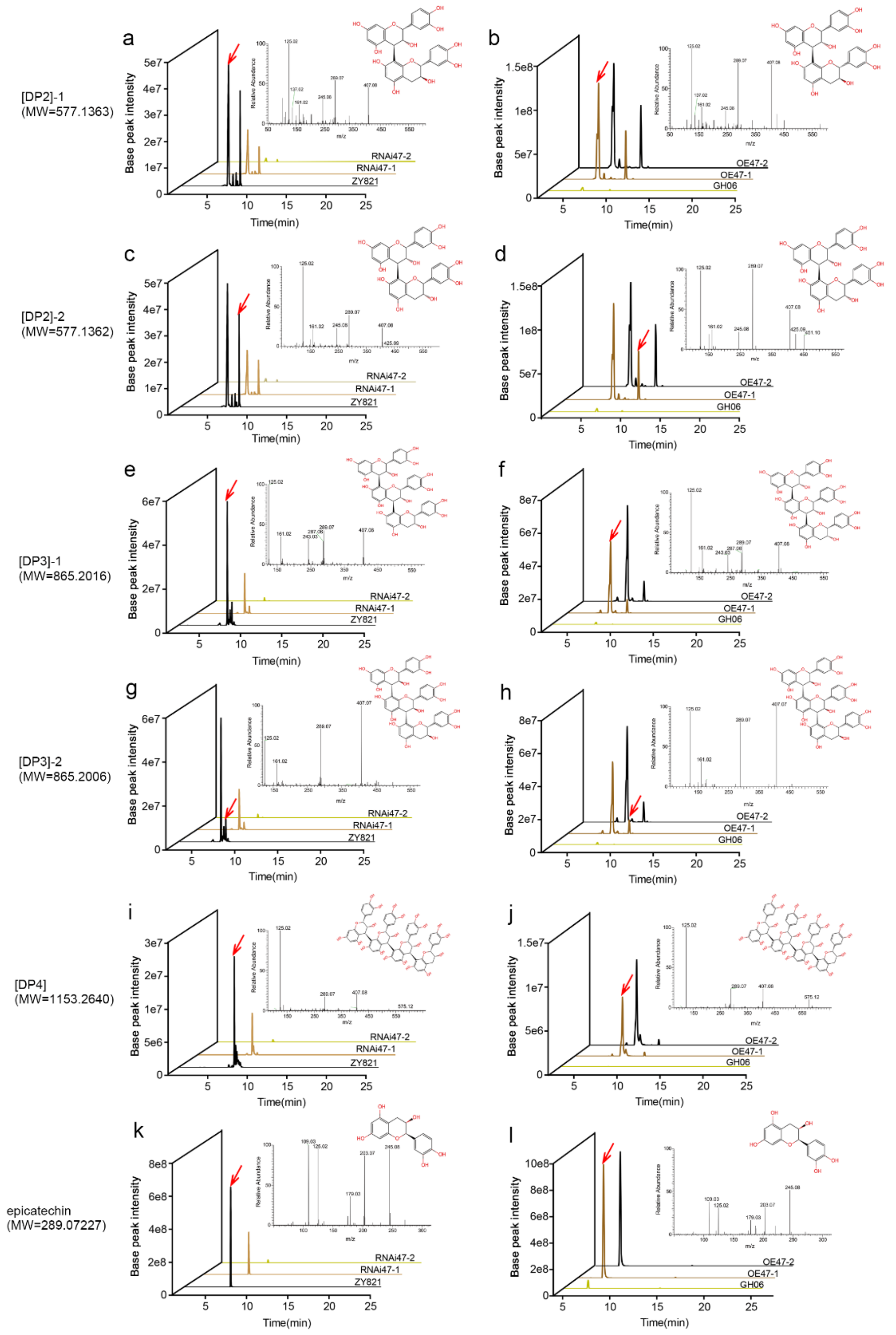

Supplementary Fig. 21. UPLC-HESI-MS/MS chromatograms of significant metabolites detected in seed coats of control plants and transgenic lines. a and **b**, [DP2]-1; **c** and **d**, [DP2]-2; **e** and **f**, [DP3]-1; **g** and **h**, [DP3]-2; **i** and **j**, [DP4]; **k** and **l**, epicatechin. Control plants, ZY821 and GH06. Transgenic lines, RNAi47-1, RNAi-*BnA09myb47a-1*; RNAi47-2, RNAi-*BnA09myb47a-2*; OE47-1, OE-*BnA09MYB47a-1*; OE47-2, OE-*BnA09MYB47a-2*.

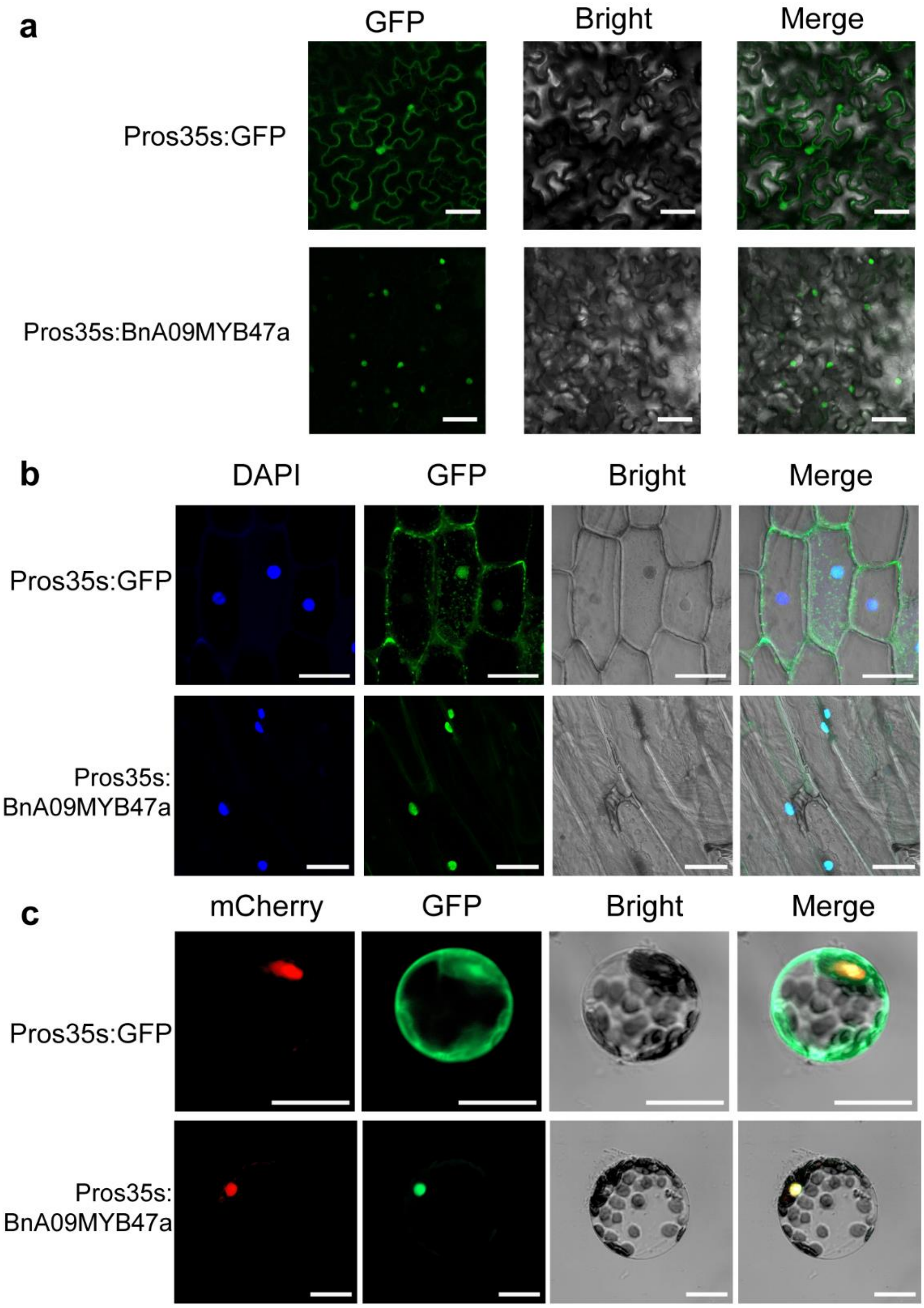

Supplementary Fig. 22. Subcellular localization analysis of BnA09MYB47a. a, Subcellular localization of free GFP and BnA09MYB47a–GFP fusion proteins in *N. benthamiana*. Scale bars, 50 μm. **b**, Subcellular localization of free GFP and BnA09MYB47a–GFP fusion proteins in epidermal cells of onion. Scale bars, 100 μm. **c**, Subcellular localization of free GFP and BnA09MYB47a–GFP fusion proteins in *Arabidopsis thaliana* protoplasts. Scale bars, 20 μm. Assays involved in **a**, **b**, and **c** were performed three times using tissues from three independent experiments.

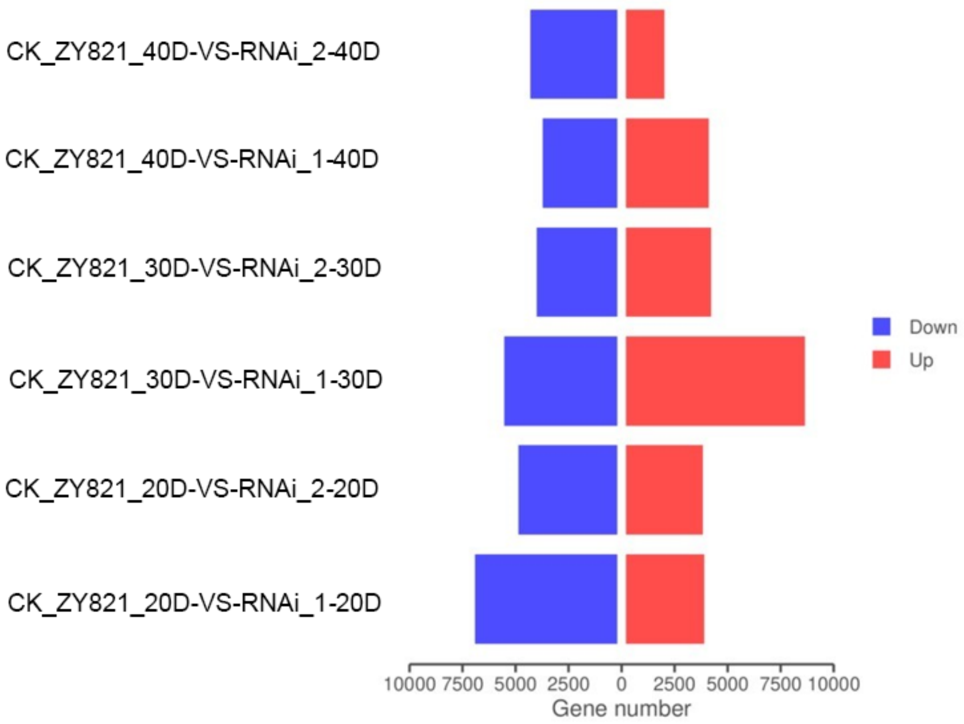

Supplementary Fig. 23. Statistics of differentially expressed genes (DEGs). X axis represents the number of screened DEGs and Y axis represents pairwise comparisons. Blue bar denotes the down-regulated genes and red bar means the up-regulated genes. There were 28,757 DEGs in total. RNAi-1, RNAi-*BnA09myb47a-1*; RNAi-2, RNAi-*BnA09myb47a-2*.

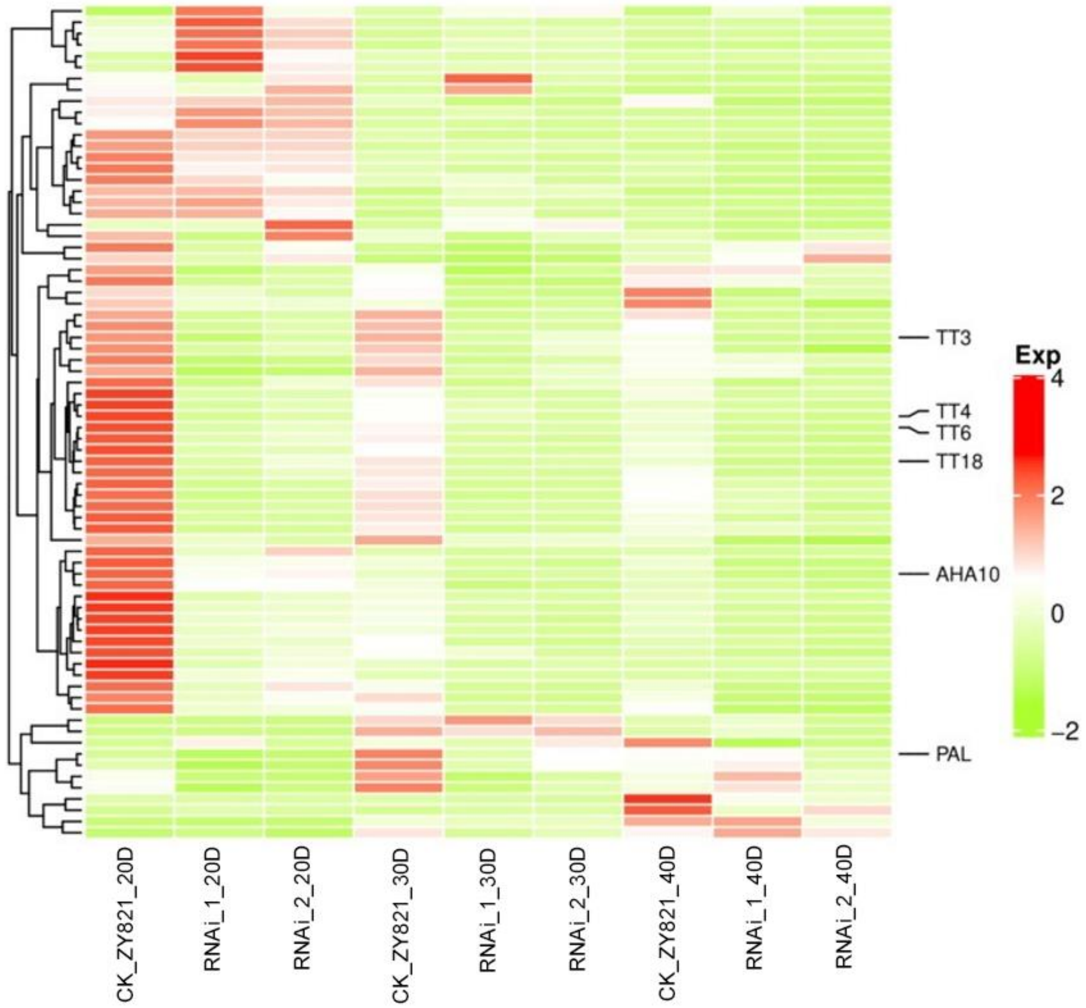

Supplementary Fig. 24. Heatmap of 74 DEGs involved in the flavonoid biosynthesis pathway. The heatmap was generated using the Complex Heatmap package where color was scaled by rows. There were 98 genes involved in flavonoid biosynthesis in total, according to the OrthoMCL cluster result, of which 74 genes were DEGs in the seed coats of RNAi-*BnA09myb47a* lines and ZY821. RNAi-1, RNAi-*BnA09myb47a-1*; RNAi-2, RNAi-*BnA09myb47a-2*. Source data are provided as a Source Data file.

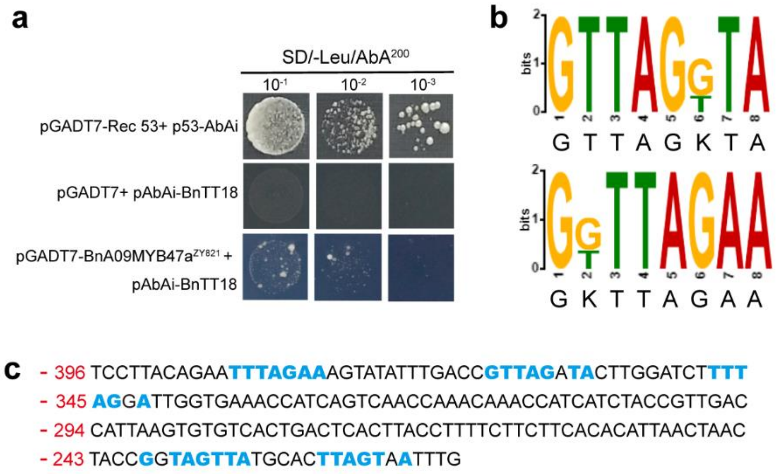

Supplementary Fig. 25. Identification of BnA09MYB47a binding targets. a, Physical interaction between BnA09MYB47a^{ZY821} and the promoter of *BnTT18* as demonstrated by a yeast one-hybrid assay. pGADT7-Rec53 and p53-AbAi were used as the positive controls. **b**, Enriched DNA motif identified in BnA09MYB47a binding sites through DAP-seq. **c**, BnA09MYB47a binding motifs in the *BnTT18* promoter.

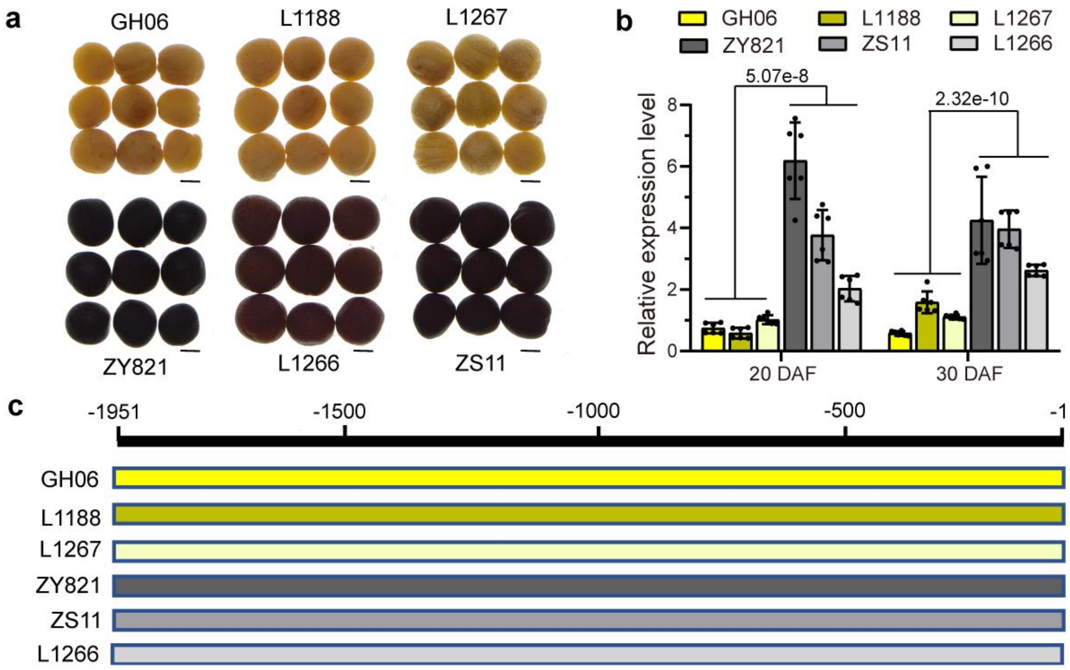

Supplementary Fig. 26. Phenotypes and characterization of *BnTT18*. (**a**) Phenotypes of *B. napus* with different genetic background. Scale bars, 2 mm. (**b**) The expression levels of *BnTT18* in developing seed coats in different genetic backgrounds. Data are presented as means ± SD (n = 6 biological replicates). *P* values were calculated using the two-tailed student's *t*-test, compared to levels in black-seed lines. (**c**) Multiple alignments of the *BnTT18* sequence. Source data are provided as a Source Data file.
