# Supplementary material for: Comparative genomic analyses reveal the genetic basis of the yellow-seed trait in Brassica napus
Source: Nat Commun. 2023 Aug 25;14:5194. doi: 10.1038/s41467-023-40838-1 (PMC10457299; doi:10.1038/s41467-023-40838-1)
Supplement: Supplementary file 2 — Description of Additional Supplementary Files [file 41467_2023_40838_MOESM2_ESM.pdf]

Description of Additional Supplementary Files

File Name: Supplementary Data 1
Description: Statistics of PacBio data.

File Name: Supplementary Data 2
Description: Statistics of Illumina data.

File Name: Supplementary Data 3
Description: Comparison of B. napus assemblies.

File Name: Supplementary Data 4
Description: Statistics of structural variations between ZY821 and GH06.

File Name: Supplementary Data 5
Description: General statistics of repeats in the GH06 and ZY821 genomes.

File Name: Supplementary Data 6
Description: Transposable element (TE, interspersed repeat) contents in the assembled GH06 and ZY821 genomes.

File Name: Supplementary Data 7
Description: Number of functional annotations for predicted genes of the B. napus assemblies.

File Name: Supplementary Data 8
Description: General statistics of the predicted protein-coding genes.

File Name: Supplementary Data 9
Description: Non-coding RNAs in the GH06 and ZY821 assemblies.

File Name: Supplementary Data 10
Description: Phenotypic analysis of seed coat colors of B. napus in different environments for the recombinant inbred lines and the parents.

File Name: Supplementary Data 11
Description: Sequencing data statistics of the QTL-seq experiment.

File Name: Supplementary Data 12
Description: Mapping rates with different reference genomes.

File Name: Supplementary Data 13
Description: SNP and InDel annotation results of the QTL-seq experiment.

File Name: Supplementary Data 14
Description: Results of mapping the QTL for seed coat color as detected by the CIM method in different environments.

File Name: Supplementary Data 15

Description: Locations of the QTL markers in the Darmor-bzh (v4.1), GH06 and ZY821 genomes.

File Name: Supplementary Data 16
Description: Locations of the InDel markers in the Darmor-bzh (v4.1), GH06 and ZY821 genomes.

File Name: Supplementary Data 17
Description: Candidate genes and annotations in the Darmor-bzh (v4.1), ZY821 and GH06 genomes.

File Name: Supplementary Data 18
Description: Organism name and gene accession numbers for phylogenetic analysis.

File Name: Supplementary Data 19
Description: The main differential flavonoid metabolites identified by UPLC-HESI-MS/MS in developing B. napus seed coats.

File Name: Supplementary Data 20
Description: Comparisons of the differentially accumulated flavonoid metabolites (μg/g FW) between ZY821 and RNAi-BnA09myb47a lines during different developmental stages.

File Name: Supplementary Data 21
Description: Comparisons of differentially accumulated flavonoid metabolites (μg/g FW) between GH06 and BnA09MYB47a^{ZY821}-overexpression lines during different developmental stages.

File Name: Supplementary Data 22
Description: Primers used in this study.
