# Supplementary material for: Comparative genomic analyses reveal the genetic basis of the yellow-seed trait in Brassica napus
Source: Nat Commun. 2023 Aug 25;14:5194. doi: 10.1038/s41467-023-40838-1 (PMC10457299; doi:10.1038/s41467-023-40838-1)
Supplement: Supplementary file 25 — Reporting Summary [file 41467_2023_40838_MOESM25_ESM.pdf]

nature portfolio

Corresponding author(s): Jiana Li

Last updated by author(s): Jul 29, 2023

Reporting Summary

Nature Portfolio wishes to improve the reproducibility of the work that we publish. This form provides structure for consistency and transparency in reporting. For further information on Nature Portfolio policies, see our Editorial Policies and the Editorial Policy Checklist.

Statistics

For all statistical analyses, confirm that the following items are present in the figure legend, table legend, main text, or Methods section.

n/a	Confirmed	
☐	☒	The exact sample size (*n*) for each experimental group/condition, given as a discrete number and unit of measurement
☐	☒	A statement on whether measurements were taken from distinct samples or whether the same sample was measured repeatedly
☐	☒	The statistical test(s) used AND whether they are one- or two-sided<br>*Only common tests should be described solely by name; describe more complex techniques in the Methods section.*
☒	☐	A description of all covariates tested
☒	☐	A description of any assumptions or corrections, such as tests of normality and adjustment for multiple comparisons
☒	☐	A full description of the statistical parameters including central tendency (e.g. means) or other basic estimates (e.g. regression coefficient) AND variation (e.g. standard deviation) or associated estimates of uncertainty (e.g. confidence intervals)
☐	☒	For null hypothesis testing, the test statistic (e.g. *F*, *t*, *r*) with confidence intervals, effect sizes, degrees of freedom and *P* value noted<br>*Give P values as exact values whenever suitable.*
☒	☐	For Bayesian analysis, information on the choice of priors and Markov chain Monte Carlo settings
☒	☐	For hierarchical and complex designs, identification of the appropriate level for tests and full reporting of outcomes
☒	☐	Estimates of effect sizes (e.g. Cohen's *d*, Pearson's *r*), indicating how they were calculated

Our web collection on statistics for biologists contains articles on many of the points above.

Software and code

Policy information about availability of computer code

Data collection	PacBio RSII, PacBio SMRT-Analysis, IrysSolve, Illumina NovaSeq 6000 platform. Near-Infrared Reflectance Analyzer (DS2500, Foss Analytical A/S)
Data analysis	flye(version 2.5) , pillon (v1.22), HiC-Pro (V2.11.1), Juicer (V1.6.2), JuiceBox (v1.11.8), and 3D-DNA (version 180114) for genome assembly, Tandem Repear Finder (TRF, version 4.07), RepeatModeler (v1.08), LTR_FINDER (v1.0.6), RepeatMasker (version 4.0.7) and RepeatProteinMasker for repeats annotation. TblastN (version 2.25), BLASTP (version 2.25), BLAST (version 2.2.24), GeneWise (version 2.4.1), Augustus (version 3.2.1), GlimmerHMM (version 3.0.4), SNAP (version 2006-07-28), hisat2 (verion 2.0.1), Stringtie (version 1.2.2), InterProScan (V5.30), TransDecoder (version 3.0.1) and EvidenceModeler (EVM) for gene annotation. tRNAscan-SE (version 1.3.1), BlastN (version 2.2.24), Rfam database (release 12.0) and INFERNAL (version 1.1.1) were used for Annotation of non-coding RNAs. Trimmomatic (version 0.36) for filtering raw sequencing data. BWA (Version: 0.7.12) for reads alignment, Samtools (Version: 1.3.1) and GATK (version 3.7-0-gcfedb67) for SNP and INDEL detection. Annovar (version: 2013-06-21) for SNP and INDEL annotation. Phenotype data statistical analyses were performed using SPSS 13.0 and GraphPad Prism 8.0.1 software. Other softwares have been well described in the method. MSTmap and Joinmap (version 4.0) were used for linkage map construction. Windows QTL Cartographer (version 2.5) was used for QTL analysis and the results were displayed by Mapchart (version 2.1).

For manuscripts utilizing custom algorithms or software that are central to the research but not yet described in published literature, software must be made available to editors and reviewers. We strongly encourage code deposition in a community repository (e.g. GitHub). See the Nature Portfolio guidelines for submitting code & software for further information.

Data

Policy information about availability of data

All manuscripts must include a data availability statement. This statement should provide the following information, where applicable:

- Accession codes, unique identifiers, or web links for publicly available datasets
- A description of any restrictions on data availability
- For clinical datasets or third party data, please ensure that the statement adheres to our policy

The genome sequence and annotation data for ZY821 and GH06 and their transcriptomic data have been deposited in the NCBI database under BioProject accession PRJNA770894. Seeds of accessions used, phenotype data, and transcriptome datasets generated and analyzed in the current study are available from the corresponding authors on reasonable requests.

Human research participants

Policy information about studies involving human research participants and Sex and Gender in Research.

Reporting on sex and gender	not applicable
Population characteristics	not applicable
Recruitment	not applicable
Ethics oversight	not applicable

Note that full information on the approval of the study protocol must also be provided in the manuscript.

Field-specific reporting

Please select the one below that is the best fit for your research. If you are not sure, read the appropriate sections before making your selection.

☒ Life sciences ☐ Behavioural & social sciences ☐ Ecological, evolutionary & environmental sciences

For a reference copy of the document with all sections, see nature.com/documents/nr-reporting-summary-flat.pdf

Life sciences study design

All studies must disclose on these points even when the disclosure is negative.

Sample size	For whole-genome sequencing of a bulked DNA study, 188 recombinant inbred lines was constructed by crossing GH06 (yellow-seeded) and ZY821 (black-seeded) after 11 generations of selfing. Twenty-five individual lines with extreme yellow-seeded traits and 25 individual lines with extreme black-seeded traits were selected for developing two extreme bulks. According to previous studies these sample sizes are sufficient. For example, In the study of Huang, X., et al (2009) the used 150 RILs for QTL analysis. In the study of Takagi, H., et al. (2013) , they sampled DNA from 10 to 20 individuals from each extremity bulk for QTL-seq analysis.<br>Huang, X., et al. (2009). "High-throughput genotyping by whole-genome resequencing." Genome Res 19(6): 1068-1076.<br>Takagi, H., et al. (2013). "QTL-seq: rapid mapping of quantitative trait loci in rice by whole genome resequencing of DNA from two bulked populations." Plant J 74(1): 174-183.
Data exclusions	No data were excluded.
Replication	individual lines with extreme traits were selected, so replication not relevant to bulked DNA study. Three independent biological replicates for each samples were subjected to illumina RNA-seq. All attempts at replication were successful for 'illumina RNA-seq' experiment.
Randomization	For QTL-seq, the extreme bluk samples are selected as the most extreme yellow or black seeds samples.
Blinding	Blinding was not relevant to this study because it did not involve a clinical trial.

Reporting for specific materials, systems and methods

We require information from authors about some types of materials, experimental systems and methods used in many studies. Here, indicate whether each material, system or method listed is relevant to your study. If you are not sure if a list item applies to your research, read the appropriate section before selecting a response.

Materials & experimental systems

n/a	Involved in the study	
☐	☒	Antibodies
☒	☐	Eukaryotic cell lines
☒	☐	Palaeontology and archaeology
☒	☐	Animals and other organisms
☒	☐	Clinical data
☒	☐	Dual use research of concern

Methods

n/a	Involved in the study	
☒	☐	ChIP-seq
☒	☐	Flow cytometry
☒	☐	MRI-based neuroimaging

Antibodies

Antibodies used	Monoclonal ANTI-FLAG M2, Affinity Purified (Product Code F 1804). For highly sensitive and specific detection of FLAG fusion proteins by WB, IP, IF, immunohistochemistry and immunocytochemistry. Optimized for single band detection of FLAG fusion proteins in mammalian, plant and bacterial expression systems.
Validation	Affinity-Purified ANTI-FLAG® M2 Antibody with High Specificity and Sensitivity<br>Michael C. Harvey, John G. Dapron, Richard J. Mehigh, Ned Watson, Heidi Heimlich, Dan Allison, Kelly Foster, Jeffrey J. Porter, Tom Juehne, Beth K. Ewing, Jessica M. Moeller, Dian Er Chen, William K. Kappel, and Graham B.I. Scott.<br>Peer-reviewed papers:<br>Histone H1 Limits DNA Methylation in Neurospora crassa. Michael Seymour. G3 (Bethesda, Md.) (2016).<br>ADAR2 regulates RNA stability by modifying access of decay-promoting RNA-binding proteins. Aparna A. Nucleic Acids Research, 45 (7), 4189-4201 (2017).
